# Study protocol for safety and efficacy of all-oral shortened regimens for multidrug-resistant tuberculosis: a multicenter randomized withdrawal trial and a single-arm trial [SEAL-MDR]

**DOI:** 10.1186/s12879-023-08644-8

**Published:** 2023-11-27

**Authors:** Liang Fu, Juan Xiong, Haibo Wang, Peize Zhang, Qianting Yang, Yi Cai, Wenfei Wang, Feng Sun, Xilin Zhang, Zhaoqin Wang, Xinchun Chen, Wenhong Zhang, Guofang Deng

**Affiliations:** 1 Division Two of Pulmonary Diseases Department, Shenzhen Third People’s Hospital, Shenzhen Clinical Research Center for Tuberculosis, National Clinical Research Center for Infectious Disease (Shenzhen), Southern University of Science and Technology, 29 Bulan Rd, Longgang District, Shenzhen, 518112 China; 2 Health Science Center, Shenzhen University, 3688 Nanhai Avenue, Nanshan District, Shenzhen, 518060 China; 3 Peking University Clinical Research Institute, Peking University First Hospital, Xueyuan Rd 38#, Haidian District, Beijing, 100000, 100191 China; 4 Department of Pathogen Biology, Guangdong Key Laboratory of Regional Immunity and Diseases, Shenzhen University School of Medicine, 1066 Xueyuan Ave, Nanshan District, Shenzhen, 518060 China; 5 Department of Infectious Diseases, Shanghai Key Laboratory of Infectious Diseases and Biosafety Emergency Response, National Medical Center for Infectious Diseases, Huashan Hospital, Fudan University, 12 Urumqi Middle Road, Jing’an District, Shanghai, 200040 China; 6 Tuberculosis Prevention and Control Department, The Fourth People’s Hospital of Foshan, 106 Jinlannan Rd, Chancheng District, Foshan, 528000 China

**Keywords:** Multidrug-resistant, Tuberculosis, Efficacy, Safety, Trial

## Abstract

**Introduction:**

The urgent need for new treatments for multidrug-resistant tuberculosis (MDR-TB) and pre-extensively drug-resistant tuberculosis (pre-XDR-TB) is evident. However, the classic randomized controlled trial (RCT) approach faces ethical and practical constraints, making alternative research designs and treatment strategies necessary, such as single-arm trials and host-directed therapies (HDTs).

**Methods:**

Our study adopts a randomized withdrawal trial design for MDR-TB to maximize resource allocation and better mimic real-world conditions. Patients’ treatment regimens are initially based on drug resistance profiles and patient’s preference, and later, treatment-responsive cases are randomized to different treatment durations. Alongside, a single-arm trial is being conducted to evaluate the potential of sulfasalazine (SASP) as an HDT for pre-XDR-TB, as well as another short-course regimen without HDT for pre-XDR-TB. Both approaches account for the limitations in second-line anti-TB drug resistance testing in various regions.

**Discussion:**

Although our study designs may lack the internal validity commonly associated with RCTs, they offer advantages in external validity, feasibility, and ethical appropriateness. These designs align with real-world clinical settings and also open doors for exploring alternative treatments like SASP for tackling drug-resistant TB forms. Ultimately, our research aims to strike a balance between scientific rigor and practical utility, offering valuable insights into treating MDR-TB and pre-XDR-TB in a challenging global health landscape. In summary, our study employs innovative trial designs and treatment strategies to address the complexities of treating drug-resistant TB, fulfilling a critical gap between ideal clinical trials and the reality of constrained resources and ethical considerations.

**Trail registration:**

Chictr.org.cn, ChiCTR2100045930. Registered on April 29, 2021.

**Supplementary Information:**

The online version contains supplementary material available at 10.1186/s12879-023-08644-8.

## Introduction

Tuberculosis (TB) remains a pressing global health issue with substantial mortality [1, 2]. The emergence of multidrug-resistant (MDR-TB) and pre-extensive drug-resistant TB (pre-XDR-TB) exacerbates the problem, contributing to significant morbidity [3, 4], mortality (30–50%) [5, 6], and economic burden. Globally, approximately 500,000 MDR/RR-TB cases arise annually, with pre-XDR-TB constituting around 26% of this number [3].

Recent individual patient data (IPD) meta-analysis points to the efficacy of specific second-line drugs like linezolid (Lzd), fluoroquinolones (FQs), bedaquiline (Bdq), clofazimine (Cfz), and others in treating MDR-TB [7]. WHO and other agencies have subsequently devised treatment guidelines that categorize these second-line drugs into three groups (A, B, C), based on their efficacy and safety [8, 9]. For example, The 2020 and 2022 WHO guidelines, favor a 6-month regimen of Bdq, pretomanid (Ptm), Lzd, and moxifloxacin (Mfx) (BPaLM), or a 9-month regimen of seven drugs, over conventional 18-month individualized regimens [10, 11]. Despite these advancements, evidence for modifying WHO-recommended short regimens is limited, underscoring the need for tailored, shorter treatment protocols for MDR-TB that are grounded in local clinical realities and evidence-based decision-making [12].

China, an upper-middle-income country, bears a significant MDR-TB burden but lacks comprehensive studies addressing these issues [13]. China has the world’s second-largest MDR/RR-TB population, with 33,000 new cases in 2021 and a treatment success rate of only 53%, below the global average of 60% [1]. Our previous multicenter cohort study demonstrated the efficacy of three 9-month, all-oral regimens in treating MDR-TB and pre-XDR-TB, significantly improving the treatment course and outcomes [14]. Regimen A (Bdq + Lzd + Mfx + Cs + Pza) and Regimen B (Lzd + Mfx + Cs + Cfz + Pza) served as treatments for MDR-TB patients, allocated to Groups A and B based on patient preference. Meanwhile, Regimen C (Bdq + Lzd + Cs + Cfz + Pza) was designated for pre-XDR-TB patients. In addition to these conventional chemotherapies, our team has focused on a host-directed therapy, salazosulfapyridine (SASP). Regimen D (Lzd + Cs + Cfz + Pza + SASP) had a satisfactory efficacy and safety for pre-XDR-TB treatment (not published yet).

Building on our prior work, we aim to conduct a randomized controlled trial to further optimize treatment durations without revisiting drug combinations. This study protocol (date 2022-07-01, version 1.5) outlines our methodology for achieving this goal.

## Methods

### Trial design and oversight

**Fig. 1:**
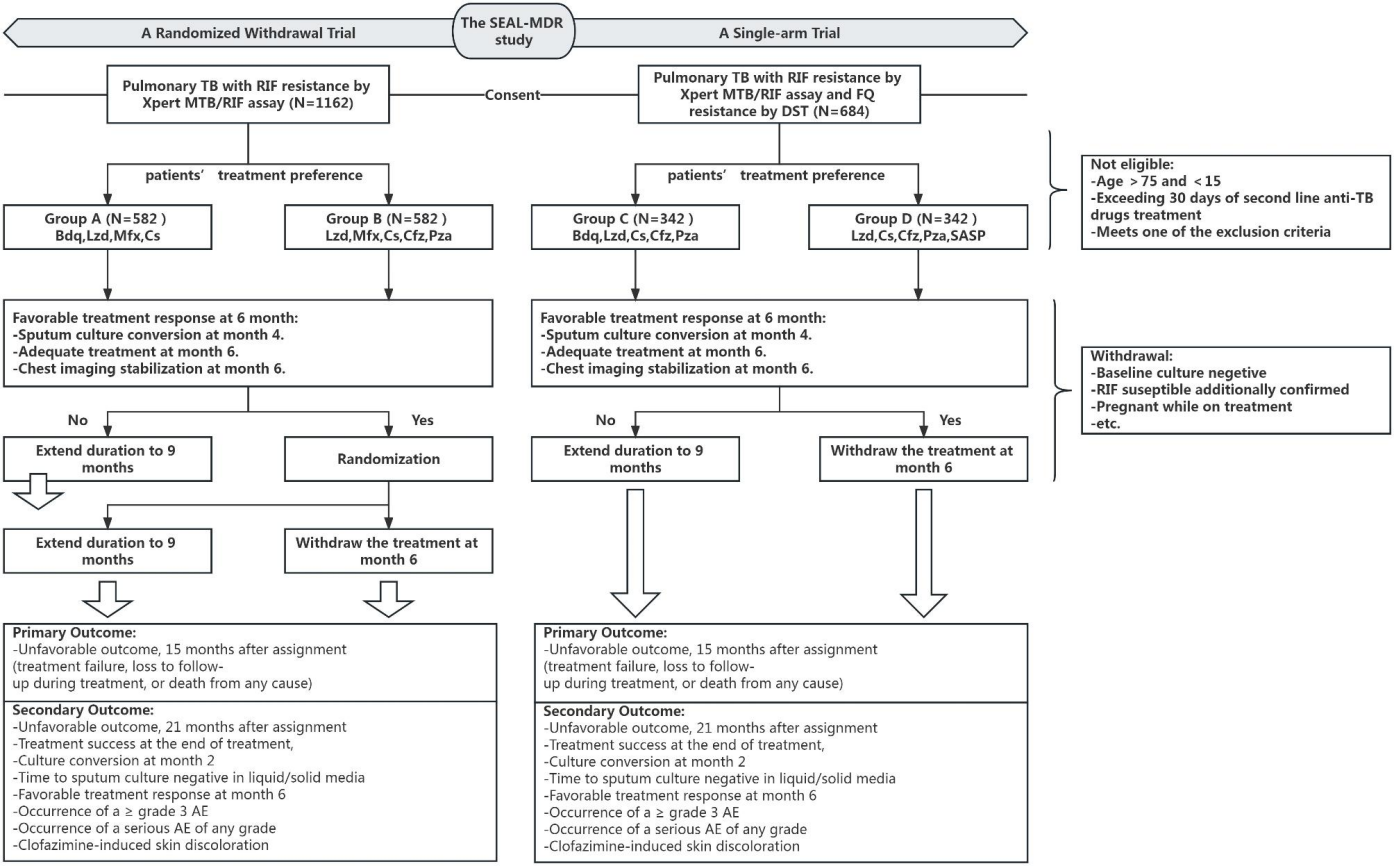
The flow diagram of the SEAL-MDR study. The diagram outlines the key steps in the study, starting with patient recruitment, followed by initial selection of treatment regimens based on *M. tuberculosis* drug resistance spectrum and patient preferences. For the randomized withdrawal trial, patients with a favorable treatment response will be randomized to 6 or 9 months of treatment after 6 months of treatment. Participants with a poor treatment response will not be randomized but will continue on a 9-month treatment regimen. In the single-arm trial, participants with a favorable response will stop treatment at month 6, otherwise treatment will be extended to 9 months. Throughout the study, FQ resistance results may be obtained at various stages to guide initial and subsequent regimen adjustments. Participants will attend visits at 12 months post-treatment to obtain primary and secondary endpoints. (Abbreviations in this figure: Bdq: bedaquiline; Cfz: clofazimine; DST: Drug susceptibility testing; FQ: fluoroquinolone; Lzd: linezolid; Mfx: moxifloxacin; Pza: Pyrazinamide; Rif: rifampicin; SASP: Sulfasalazine; TB: tuberculosis)

We are conducting a two-part, patient-stratified, treatment-response-guided study: a randomized withdrawal trial for MDR-TB and a single-arm trial for pre-XDR-TB (Fig. 1; Table 1). For MDR-TB, patients are assigned to either Regimen A (Bdq, Lzd, Mfx, Cs) or Regimen B (Lzd, Mfx, Cs, Cfz, Pza) based on treatment preference and FQ drug susceptibility tests (DST). Compared to our previous study, Pza is deleted from Regimen A. For pre-XDR-TB, we employ Regimen C (Bdq, Lzd, Cs, Cfz, Pza) and Regimen D (Lzd, Cs, Cfz, Pza, SASP). Each regimen lasts 6–9 months. Post-6 months, participants in Groups A and B are subdivided into 6-month and 9-month treatment durations based on randomization, while those in Groups C and D are similarly divided based on clinical judgment. For the randomized withdrawal trial, the primary hypothesis is that the 6-month regimen will be non-inferior to the 9-month regimen in terms of unfavorable outcomes.

**Table 1:** Trial regimens

FQ susceptibility	Regimen	Drugs	Durations
MDR-TB	A	bedaquiline, linezolid, moxifloxacin, cycloserine and pyrazinamide	6–9 months
	B	linezolid, moxifloxacin, cycloserine, clofazimine and pyrazinamide	6–9 months
Pre-XDR-TB	C	bedaquiline, linezolid, cycloserine, clofazimine and pyrazinamide	6–9 months
	D	linezolid, cycloserine, clofazimine, pyrazinamide and salazosulfapyridine	6–9 months

This study, named SEAL-MDR, was registered with the Chinese Clinical Trials Registry and has received ethical approval from participating hospitals. Written informed consent will be obtained from all adult participants or their legal guardians by trained medical personnel who may be an investigator, clinician, or designated nurse experienced in explaining the elements of clinical trials.

### Study sites

Trial sites should have adequate staffing, facilities, and recruitment capabilities. They must also have access to quality-assured laboratories capable of performing required tests and have secure data management systems in place. Thirty-two sub-centers are participating, with competitive enrollment until required sample sizes are met (Table S8). The four treatment regimen groups A-D may be unevenly distributed in each sub-center, and competitive enrollment is used in this clinical trial until the sample size meets the requirements for each group.

### Selection of participants

Eligible participants are 15–75 years old with confirmed pulmonary MDR-TB or pre-XDR-TB, subject to exclusion criteria, which include QTcF ≥ 450 ms, HIV positivity, known drug allergies, and abnormal lab values among others. Full inclusion and exclusion criteria are outlined in Table S1.

Before trial-specific procedures, eligible patients are given comprehensive information, including known risks and potential benefits such as reduced treatment duration and costs. Consent is documented, and screening logs are securely maintained.

### Confirmation of MDR/Pre-XDR-TB

DST is a key prerequisite for trial entry, with varying methodologies dependent on local lab capabilities. Mandatory DST results are required for rifampicin, isoniazid, and FQs. Other study drugs like Bdq, Lzd, Cfz, and Pza are not obligatory for DST. Patients with resistance to these non-mandatory drugs are excluded from the study.

For DST, rifampicin resistance is confirmed using the Xpert MTB/RIF assay (Cepheid, USA), while susceptibility to FQs, isoniazid, and ethambutol is determined either through genotypic (gDST) or phenotypic (pDST) assays measuring minimum inhibitory concentration (MIC) (Thermo Scientific, USA), with genotypic methods including PCR melting curve analysis (Zeesan Biotech, China), whole-genome sequencing (Novogene, China), and mass spectrometry (Conlight Medical, China).

### Randomization, enrolment and follow-up

Before initiating treatment, all available DST results (e.g., Xpert, gDST, pDST) are employed to triage patients with MDR-TB and pre-XDR-TB. Considering variables such as regimen affordability and past adherence, MDR-TB patients are allocated to either Regimen A or B (Table 1). Regimen B is preferred for uninsured participants, those concerned about drug costs, and individuals willing to accept potential skin discoloration caused by Cfz. Pre-XDR-TB patients are allocated to either Regimen C or D, with Regimen D being better suited for those without insurance coverage and lesser financial means.

Should post-entry DST results confirm resistance to any study drug, treatment regimens will be adjusted according to established clinical practice principles in our previous studies [1]. For example, If initial gDST is unavailable and later pDST reveals FQ resistance 2–3 months post-enrollment, participants will transition from Regimens A/B to C/D, incorporating the prior treatment duration into the revised 6-month course. In certain scenarios, participant withdrawal may be necessitated. For example, participants unwilling to switch sill be prescribed long-course treatment and excluded from the study.

Participants initially undergo a 6-month treatment course. A blinded mid-point review committee evaluates clinical events and favorable treatment responses during the sixth-month visit (FR-6, Table S2). This assessment includes sputum culture conversion at month four, adequate drug intake at month six (excluding those who received less than ~ 90% of study doses), and chest computed tomography (CT) stabilization. Participants having a FR-6 will be centrally randomized into either a 6- or 9-month treatment group via MedSci RTSM Cloud Platform (rtsm.medsci.cn). Otherwise, they will continue their current regimen until month nine. Failure to convert by the eighth month necessitates an extension to an 18–20 month regimen, marking it as a treatment failure.

Participants attend baseline screenings, bi-weekly visits for the first two months, monthly visits until treatment completion, and quarterly check-ups during a 12-month post-treatment period (Tables S3 and S4). The screening procedures include medical history, physical examination, vision tests, peripheral neuropathy screening, psychological assessments, and the collection of various samples for testing. CT scans are preferred over X-rays for assessing lung lesions, which is a routine at the study sites.

Upon passing the screening, eligible participants are enrolled, registered offline, and assigned a treatment regimen by an authorized researcher at each sub-center. These assignments are re-verified by the Central Coordination and Quality Control Group (CCQCG) at Shenzhen Third People’s Hospital.

Effectiveness and safety metrics are evaluated at each follow-up visit through symptom checklists, clinical exams, and ongoing diagnostic tests. Grade 3 or 4 clinical and laboratory adverse events (AEs) are defined per the DAIDS AE Grading Table [12]. Chest CT scans are regularly updated and reviewed blindly by two independent experts using a standardized approach (Table S2). An endpoint review committee examines unfavorable clinical outcomes like treatment failure, TB recurrence, or death. Adherence metrics and educational counseling are also provided, along with psychological support through WeChat and telephone channels. Post-treatment, successfully treated cases will be monitored for 12 months to assess any TB recurrence.

### Treatment of patients

Details of the drug combinations and duration for each of the Regimens A-D are shown in Table 1. Table 2 provides details on the drug types and dosages included in each regimen. In case of grade 3 or 4 AE triggered by Lzd, the Lzd dose was cut to 300 mg or ceased entirely as required. The rest of the five drugs, Bdq included, were given throughout the course of the treatment. All medications used in the study have received approval from the National Medical Products Administration and are prescribed solely by authorized study physicians at the respective research sites. None of the medications are provided free of charge. Contrary to employing the Directly Observed Treatment (DOT) strategy, this study designates a clinic staff member to oversee each patient’s treatment. This individual is tasked with verifying medication consumption, documenting it on the treatment card, and providing guidance to ensure proper administration and patient adherence.

**Table 2:** Trial regimen drugs and doses

Drug	Weight group		Usage
	≤ 50 kg	> 50 kg	
Bedaquiline	400 mg daily for 2 weeks followed by 200 mg three times a week		Once, with meal
Linezolid	600 mg daily		Once, before or after meal
Moxifloxacin	400 mg daily		Once, before or after meal
Levofloxacin	500 mg daily	750 mg daily	Once, before or after meal
Cycloserine	500 mg daily	750 mg daily	Divided in two doses, before or after meal
Clofazimine	100 mg daily		Once, with meal
Pyrazinamide	1500 mg daily		Once, before or after meal
Sulfasalazine	3000 mg daily		Divided in three doses, with meal

Based on our prior cohort study experience and international guidelines, we have formulated a manual for the management of adverse reactions to second-line anti-TB drugs for the SEAL-MDR study (Table S5). Management of treatment interruptions was shown in Table S6.

### Outcome measures

In this study, outcomes are categorized at the end of a 12-month post-treatment follow-up. Participants fall into one of three categories: unfavorable outcome, favorable outcome, or not assessable - the latter are excluded from the primary analysis (Table S7). The primary efficacy outcome, determined 15 months after initial assignment, includes treatment failure, loss to follow-up, or death from any cause. Treatment failure is a multifaceted term, defined by the end-point review committee, and encompasses lack of clinical and/or bacteriological response, adverse drug reactions that induced regimen adjustments, or newly identified drug resistance. Specifically, bacteriologic failure is defined by the presence of two genetically identical, *M. tuberculosis*-culture-positive sputum samples within the month 8 visit window. Bacteriologic relapse and reinfection refer to a positive test for a genetically identical or different *M. tuberculosis* strain, respectively, within 12 months post-treatment. In cases where strain genotyping is not feasible, the term ‘bacteriologic recurrence’ is employed. Secondary efficacy outcomes include unfavorable status at 21 months post-assignment, end-of-treatment success, culture conversion by month 2, time to sputum culture-negative status in liquid media, and favorable treatment response at FR-6. Safety outcomes involve the occurrence of AEs graded ≥ 3 or serious AEs of any grade within 6–9 months of treatment initiation, as per the DAIDS AE Grading Table [15]. Clofazimine-induced skin discoloration is also specifically documented (Table S2).

### Sample size calculation

Group A/B in the randomized withdrawal trial: According to a 2018 IPD meta-analysis [7] and our previous study [14], we assumed that two groups (A and B) have an assumed favorable outcome rate of 73%. Taking into consideration various parameters like a 90% FR-6 rate, a 1:1 random assignment to two different durations (month 6 or month 9), a 15% margin of non-inferiority, a one-sided significance level of 5%, 80% power, and a 20% drop rate, 304 participants are needed for each group. Hence, 304 + 304 = 608 participants in total for groups A and B.

Calculation for Group C/D in the single-arm trial: Two other groups (C and D) have an assumed favorable outcome rate of 57% [7, 14]. Using the same parameters (15% margin of non-inferiority, a one-sided significance level of 5%, 80% power, and a 20% drop rate), 85 participants are needed for each group. Hence, 85 + 85 = 170 participants in total for groups C and D.

Combining both sets of groups, the total sample size required would be 608 + 170 = 778 participants for the SEAL-MDR study.

### Statistical analysis

Efficacy outcomes will be evaluated using modified intention-to-treat (mITT) and per protocol (PP) approaches, focusing on mITT. The safety analysis includes all participants receiving at least one dose of study drugs. The mITT group consists of those randomized and treated at least once, while the PP group comprises mITT members who complete > 80% of planned treatment and adhere to the protocol.

Analysis is performed in SPSS 26.0. Categorical data are presented as counts and percentages, and continuous data as means (standard deviations, SD) and medians (interquartile range, IQR). Depending on data distribution, Student’s t-test or non-parametric tests are used for continuous variables, and Chi-square or Fisher’s test for categorical ones. Primary analysis estimates unfavorable outcome rates in subgroups of Regimens A and B, with 95% CI for each group. Kaplan-Meier and Cox models evaluate time to unfavorable outcomes and culture conversion. Variables with *P* < 0.1 in univariate analysis and clinical significance are incorporated into a multivariate model. A *P* < 0.05 is considered significant.

### Data collection and quality management

Data is captured through a web-based case report form (e-CRF), offline/online questionnaires, and study handbooks. CCQCG oversees document provision, participant eligibility, SAEs, critical decisions, and follow-up data integrity. Post-trial, participant data is archived for further analysis, accessible only to two main investigators from Shenzhen Third People’s Hospital.

### Data access and disclosure

Principal Investigators will have full access to the final trial dataset for the purposes of data analysis and interpretation. Co-Investigators will have access to specific portions of the dataset relevant to their contributions to the study. Sponsor and Funders will not have access to individual participant data. Data Monitoring Committee will have periodic access to interim datasets for safety monitoring.

### Monitoring and supervision of the trial

The Clinical Research Institute of Peking University (CRIPU) will perform onsite visits, ensuring protocol adherence, Good Clinical Practice, participant protection, and data accuracy. A Data and Safety Monitoring Board (DSMB), consisting of two TB specialists and one statistician from CRIPU, will review data quarterly and may issue recommendations on study protocols.

### Confidentiality

Strict confidentiality is maintained for participants’ information. Paper records are secured in specialized offices, and digital files are password-protected. Access is limited to authorized personnel. The study protocol adheres to the SPIRIT checklist.

## Discussion

Tuberculosis continues to pose a significant global health burden and is a primary cause of mortality worldwide. Recent advancements in therapeutic options, notably the introduction of bedaquiline, delamanid, and pretomanid, have revitalized TB treatment protocols. Notable Phase III clinical trials, such as Study 31/A5349, SHINE and TRUNCATE-TB [16–18] for drug-susceptible tuberculosis, and STREAM stage I and II, Nix-TB, ZeNix and TB-PRACTECAL [19–23] for MDR/pre-XDR-TB, have generated encouraging data, highlighting the potential for reduced treatment durations. The updates in guidelines by the WHO and other international bodies reflect this progress.

As of 2022, WHO has restructured the classification of second-line anti-TB drugs and recommended three primary regimens for managing MDR-TB [11]. However, each regimen possesses inherent limitations, underscoring the necessity for ongoing research and refinement. China confronts distinct challenges in MDR-TB control, characterized by a high prevalence rate, limited indigenous research, prohibitive costs of drugs, and restricted access to novel therapies. Effective management mandates a focus on robust and context-specific clinical research.

Our study employs a dual design: a RCT for treatment Groups A and B, and a single-arm observational design for Groups C and D. This approach mitigates potential bias and confounding variables, establishing it as a methodological benchmark for causal inference. Previous work substantiates the safety and efficacy of our nine-month treatment protocol, addressing ethical concerns [14, 24]. Our study utilizes a randomized withdrawal trial rather than a classical RCT due to resource constraints, specifically in procuring costly second-line drugs. Initial groupings were not randomized but chosen based on drug resistance profiles and patient preferences to better mirror real-world clinical settings. At the 6-month treatment mark, only responsive patients were randomized into 6- or 9-month courses, with non-responders directly advancing to a 9-month regimen. This approach maximizes feasibility and external validity while maintaining scientific rigor, and plans for subgroup analysis are in place to ensure comprehensive data interpretation.

Single-arm trials offer advantages in mirroring real-world clinical scenarios, enhancing the generalizability of results while being cost-effective. From an epidemiological standpoint, pre-XDR-TB constitutes only about 26% of MDR-TB cases, making it challenging to meet the sample size required for a traditional RCT. Additionally, treating pre-XDR-TB is more complex than MDR-TB, with lower success rates (57% vs. 73%) [7]. In this context, it’s arguably more ethical to allow clinicians the discretion to tailor treatment durations based on clinical insight rather than random assignment. Despite these merits, single-arm trials do come with inherent limitations like potential confounding, bias, issues with establishing causality, and generally providing a lower level of evidence.

While WHO guidelines recommend routine resistance testing for FQs and other second-line anti-TB drugs in MDR-TB patients, real-world laboratory capacity often falls short. For example, only select regions in China can perform rapid molecular tests for FQ resistance, leaving others reliant on slower phenotypic DST methods like MIC. Acknowledging this gap, our study design accommodates varying timelines for FQ resistance results to inform both initial and subsequent treatment regimens. Should resistance be identified post-treatment initiation, our protocol permits single-drug substitution without necessitating an entirely new course of treatment. Although some experts may find this approach unconventional, our preliminary data and WHO opinions support its validity and need for further study. This is particularly relevant when considering that local labs may only provide FQ resistance status 2–3 months post-treatment initiation. Therefore, our study aims to address how clinicians should adapt MDR-TB treatment plans under such constraints. While our design may diverge from the rigorous controls often seen in clinical trials, it is intentionally crafted to mirror real-world clinical scenarios. This allows us to contribute to the ongoing discourse on the merits and limitations of RCTs versus real-world research, striving to find a balance between these methodologies.

The urgency for novel TB treatments is underscored by a scant drug pipeline, despite six decades of focus on directly targeting *M. tuberculosis* with new compounds. Recent trends, however, signal a pivot towards HDTs, which aim to modulate the host’s immune response rather than eliminate the bacteria directly [25]. Such approaches either boost host defenses (antimicrobial) or regulate excessive inflammatory responses (anti-inflammatory) [26], presenting a potentially effective treatment avenue for pre-XDR-TB, a form of the disease with notoriously low treatment success rates. Although preclinical investigations into HDTs are robust, clinical trials remain conspicuously missing from the research landscape. In this context, our prior work has laid the groundwork for using SASP as a potential HDT for MDR-TB, employing both cellular and animal models to elucidate its mechanism of action [27–29]. Preliminary validations of SASP’s efficacy and safety have been demonstrated through our cohort studies [24]. The next stage of our research will involve a single-arm trial that employs a SASP-containing regimen, termed “Regimen D,“ to further explore its therapeutic potential in treating pre-XDR-TB.

In conclusion, while RCTs remain the benchmark for therapeutic efficacy evaluation, their implementation is sometimes neither feasible nor ethical in every context. Single-arm trials can provide complementary perspectives, especially in real-world clinical settings. Our study exemplifies a judicious balance between scientific rigor and practical applicability, particularly pertinent to the unique challenges China faces in MDR-TB management.

### Trial status

Recruitment began at the first site in April 2021 and is expected to be completed by December 2024.

### Electronic supplementary material

Below is the link to the electronic supplementary material.

Supplementary Material 1

Supplementary Material 2

Supplementary Material 3

Supplementary Material 4

Supplementary Material 5

## Data Availability

The results of this trial will be disseminated only by presentation at academic meetings or publication in academic journals.

## Abbreviations

ADE: Adverse drug event
AE: Adverse event
AFB: Acid-fast bacilli
Bdq: Bedaquiline
CC: Culture conversion
CCQCG: Central coordination and quality control group
Cfz: Clofazimine
CI: Confidence Interval
CRF: Case report form
CT: Computed tomography
Cs: Cycloserine
DAIDS: Division of AIDS
DST: Drug susceptibility testing
DSMB: Data and safety monitoring board
FQs: fluoroquinolones
FR-6: Favorable treatment response at month 6
gDST: Genotypic drug susceptibility testing
IQR: Interquartile range
ITT: Intention-to-treat
MIC: Minimum inhibitory concentration
MTB: Mycobacterium tuberculosis
MDR-TB: Multidrug-resistant tuberculosis
pDST: Phenotypic drug susceptibility testing
pre-XDR-TB: Pre-extensive drug-resistant tuberculosis
Pza: Pyrazinamide
PP: Per protocol
QTcF: QT interval corrected by the Fridericia’s correction formula
RR-TB: Rifampicin-resistant tuberculosis
SAE: Serious adverse event
SASP: Sulfasalazine
TB: Tuberculosis
TEAEs: Treatment emergent adverse events
XDR-TB: Extensively drug-resistant tuberculosis
