# Supplementary material for: Study protocol for safety and efficacy of all-oral shortened regimens for multidrug-resistant tuberculosis: a multicenter randomized withdrawal trial and a single-arm trial [SEAL-MDR]
Source: BMC Infect Dis. 2023 Nov 27;23:834. doi: 10.1186/s12879-023-08644-8 (PMC10683225; doi:10.1186/s12879-023-08644-8)
Supplement: Supplementary file 1 — Supplementary Material 1 [file 12879_2023_8644_MOESM1_ESM.docx]

**Table S1 Inclusion and exclusion criteria**

|  | **Detailed Description** |
| --- | --- |
| **Inclusion criteria**  **(meet all the following)** | 1. Written informed consent was obtained from the participants or legal guardian of each child.  2. Between 15-75 years old.  3. Weight greater than 30kg.  4. Lung lesions confirmed by chest radiograph and suspected of pulmonary TB, including extensive TB disease.  5. RR-TB confirmed by Xpert^®^ MTB/RIF (Cepheid, Sunnyvale, CA, USA)^a^.  FQs susceptibility available by nucleic acid amplification testing, whole genome sequencing, mass spectrometry or phenotypic drug susceptibility testing.  6. A decision to treat made by an expert panel^b^.  7. Women of child-bearing potential who are not surgically sterilized must agree to practice an adequate method of contraception (barrier method, like condoms and ligation; drug contraception is not recommended).  8. Agree to stop breastfeeding if the participant is a breastfeeding woman.  9. Have an identifiable address and stay in the area for the duration of the study. |
| **Exclusion criteria**  **(meet any of the following)** | 1. Pregnant or breast-feeding.  2. Unable to take oral medications.  3. Extrapulmonary tuberculosis^c^.  4. Known resistance to any of the study medications by any DST.  5. Known allergy or intolerance to any of the study medications.  6. Received any investigational drug for more than 1 month in the past 3 months, unless DST of the drug turns out susceptible: cycloserine, clofazimine, moxifloxacin, levofloxacin, other fluoroquinolones, linezolid or bedaquiline.  7. More than one month of treatment directed against RR/MDR-TB within 6 months preceding initiation of study drugs.  8. QTcF ≥ 450 ms (one retest is allowed during the screening to reassess the eligibility), or known history of prolonged QT syndrome, or with one or more of risk factors of prolonged QT^d^.  9. Laboratory parameters done at or within 14 days prior to screening:  • Hemoglobin level of 90 g/L or less;  • Platelet count of 75,000/mm^3^ or less;  • Serum or plasma alanine aminotransferase (ALT) or aspartate aminotransferase (AST) greater than or equal to 3 times the upper limit of normal;  • Serum or plasma total bilirubin greater than or equal to 2.5 times the upper limit of normal;  • Serum or plasma creatinine level greater than or equal to 1.5 times the upper limit of normal.  10. Cirrhosis (Child class B or C by Ultrasonography).  11. Alcoholism.  12. Mental illness (e.g., epilepsy, severe depression, irritability, or other mental illness)；  13. Diabetic patients with poor blood glucose control (HbAc1 > 7.0%) and poor adherence to improve it,  14. Cancer.  15. Drug addiction.  16. HIV-positive or AIDS, or with other immunodeficiency disease.  17. Critically ill patients with short survival (e.g., no more than 4 months) as judged by the study physician, or Karnofsky score less than 50.  18. For all women who are not surgically sterilized or who do not meet the study definition of post-menopausal, a positive pregnancy test at or within seven days prior to screening.  19. Simultaneously applying drugs that may affect the efficacy of this study or contraindicated with the study drugs^e^,  20. Currently in another drug trial.  21. Other medical conditions, that, in the investigator’s judgment, make study participation not in the individual’s best interest.  22. Current or planned incarceration or other involuntary detention. |
| **Criteria for exclusion after enrollment (‘Late exclusion’)**  **(meet any of the following)^f^** | 1. Cultures collected during screening, baseline and within half month after enrollment are all negative.  2. The phenotypic DST results suggest that it is susceptible to rifampicin.  3. The participant or the legal representative of the participant requests to withdraw.  4. The participant has adverse events or cannot tolerate the research intervention, and the researcher judges that the participant is indeed unsuitable to continue the research.  5. The participant is pregnant or unable to take effective contraceptive measures. |

^a^ At least one sputum specimen positive for M. tuberculosis by Xpert MTB/RIF testing, with semiquantitative result of ‘medium’ or ‘high’ and rifamycin resistance detected.

^b^ Potential participants include: a) Treatment-naïve cases who never used second-line anti-TB drugs, while first-line drugs usage less than 2 months is allowed before the diagnosis of MDR-TB. b) Cases of treatment failure who used second-line anti-TB drugs for more than 6 months and were evaluated to have treatment failure, or who used second-line anti-TB drugs and discontinued 2 or more of the drugs for any reason. c) Cases of TB recurrence (endogenous relapse or exogenous reinfection). The comprehensive clinical evaluation should be given by the diagnosis and treatment expert group of each sub-center, under the assistance of the center expert group. An expert group should consist of 2-5 experienced physicians in tuberculosis.

^c^ PTB with or without tuberculous pleurisy/bronchial tuberculosis/mediastinal lymph node tuberculosis is considered in the the scope of PTB in this study. Extrapulmonary tuberculosis refers to suspected or documented tuberculosis involving the central nervous system and/or bones and/or joints, and/or miliary tuberculosis and/or pericardial tuberculosis. Severe extrapulmonary TB is defined as the presence of miliary TB or TB meningitis.

^d^ Risk factors include: pathological Q waves (defined as Q waves greater than 40 ms or greater than 0.4-0.5 mV in depth); ventricular pre-excitation (e.g., Wolff Parkinson-White syndrome); complete or clinically significant incomplete left Bundle branch block or right bundle branch block; second- or third-degree heart block; intraventricular conduction delay with QRS duration greater than 120 ms; bradycardia as defined by sinus heart rate less than 50 bpm; long QT syndrome personal or family history; history of heart disease, syncope (e.g., cardiac syncope, excluding syncope due to vasovagal or epilepsy), symptomatic or asymptomatic arrhythmias (except sinus arrhythmia); torsades de pointes risk factors for ventricular tachycardia (e.g., heart failure, hypokalemia, or hypomagnesemia).

^e^ Glucocorticoids, interferons, non-steroidal anti-inflammatory drugs, monoamine oxidase inhibitors (phenethyl hydrazine, different Carbofurs et al), direct or indirect sympathomimetic drugs (such as pseudoephedrine), vasopressor drugs (such as: adrenaline, norepinephrine), dopamine drugs (such as: dopamine, dobutamine), 5-HI reuptake inhibitor, a tricyclic antidepressant, a 5-HTI receptor antagonist (amitriptyline), meperidine or buspirone.

^f^ The participants have the right to withdraw from the research at any time during the trial or in any of the mentioned situations. Once the participant withdraws from the study, the investigator must record the reasons for the withdrawal in the case report form and medical record, and complete the inspection items as far as possible. All participants who withdraw from the study due to adverse reactions or abnormal clinical laboratory test results must be followed up until the participants recover or stabilize, and subsequent outcomes should be recorded.

**Table S2 Primary and secondary outcome measures**

| **Primary outcome** | **Description** |
| --- | --- |
| a) | Unfavorable Outcome: Incidence of bacteriologic failure or relapse or clinical failure through follow up until 15 months assignment. [Time Frame: 0, 0.5, 1, 1.5, 2, 3, 4, 5, 6, 7, 8 and 9 Month during the treatment period; 3, 6, 9 and 12 Month during the follow-up period] |
| **Secondary outcome** |  |
| a) | Unfavorable Outcome: Incidence of bacteriologic failure or relapse or clinical failure through follow up until 21 months assignment. [Time Frame: 0, 0.5, 1, 1.5, 2, 3, 4, 5, 6, 7, 8 and 9 Month during the treatment period; 3, 6, 9 and 12 Month during the follow-up period] |
| b) | Sputum culture conversion proportion at month 2. [Time Frame: 0, 0.5, 1, 1.5 and 2 Month during the treatment period] |
| c) | Time to sputum culture conversion after treatment start. [Time Frame: 0, 0.5, 1, 1.5, 2, 3, 4, 5, 6, 7, 8 and 9 month during the treatment period] |
| d) | Favourable treatment response at month 6 (FR-6). It should meet all the following:  a. Sputum culture conversion at month 4. It needs two consecutive cultures taken on different occasions at least 7 days apart with the last culture done within the 4-month visit, without interval positive culture and without reversion.  b. Adequate treatment at month 6. It excludes participants who have received less than approximately 90% of study doses, which defined as approximately 90% of doses within 125% of the intended duration, at least 162 doses and no more than 225 days since treatment initiation.  c. Chest imaging stabilization at month 6, defined as that two consecutive chest CTs taken on different occasions at least 2 months apart with the last CT done within the 6-month visit were interpreted by imaging experts as: (1) lesion resorbed, significantly resorbed or unchanged; (2) cavity closed, reduced or unchanged.  Notes:   \|  \| With significant absorption \| With absorption \| Unchanged \| \| --- \| --- \| --- \| --- \| \| Lesion reduction \| ≥50% \| ≥20% and＜50% \| ＜20% \| \| Cavity reduction \| Closed or disappeared \| ≥50% \| ＜50% \| |
| e) | Incidence of Treatment Emergent Adverse Events (TEAEs) presented by incidence and seriousness, including grade 3 or greater AEs and serious adverse events (SAEs) of any grade by 9 months, as well as TB related or non-TB related death. [Time Frame: 0, 0.5, 1, 1.5, 2, 3, 4, 5, 6, 7, 8 and 9] |
| f) | Clofazimine-induced skin discoloration: time of onset, severity, duration, time to return to normal skin, influencing factors. [Time Frame: 0, 0.5, 1, 1.5, 2, 3, 4, 5, 6, 7, 8 and 9 Month during the treatment period; 3, 6, 9 and 12 Month during the follow-up period] |

**Table S7 Definition of the favorable, unfavorable and not assessable outcomes**

| **Outcome** | **Description** |
| --- | --- |
| **Unfavorable outcome** | A participant will be classified as having an unfavorable outcome if any one of the following conditions is met:  • A participant will be considered to have absence of bacteriological cure if he/she has a sputum sample, obtained at or after the last treatment month (month 9) and no later than the end of the follow-up (month 21) visit window, that is MTB Culture Positive that is indistinguishable from the initial isolate, and this is confirmed by a second sample that is MTB culture positive. A second confirmatory sample, on a different day without an intervening MTB Negative culture result, is required.  a) A single positive sputum culture result in isolation, with subsequent cultures being negative, will not be considered absence of bacteriological cure.  b) Solid or liquid culture is both allowed and should be recorded clearly.  c) If results from strain analysis are inconclusive or unavailable or strain analysis can not be done, it will be assumed that strains were indistinguishable.  • Participants who had an MTB Positive culture result when last seen during or prior to the month 9 analysis visit window, whether confirmed by a second sample or not, unless determined to have been re-infected.  • Participants who die from any cause during treatment, except from violent or accidental cause (e.g. road traffic accident). Suicide during study treatment will be classified as an unfavorable outcome.  • Participants who die during the follow-up phase where the cause of death is considered related to tuberculosis.  • Participants who are withdrawn from follow-up or lost to follow-up prior to the scheduled end of treatment of study treatment, except for pregnancies and violent or accidental death that are instead classified as having a Not Assessable outcome.  • Participants receiving any one or more of the following, except when given for failure or recurrence subsequently shown to be a reinfection with a strain of MTB different from that or those identified at study entry through genotyping methods:  a) Extension of treatment beyond that permitted by the protocol; excepting  a. Temporary drug re-challenge. b. Over-treatment with drugs from assigned study kits. To be specific, treatment is extended beyond the scheduled end of treatment for any reason other than making up of days when no treatment was given (missed treatment) for a maximum of 8 weeks. A maximum of half a month (15 days) of extra treatment (irrespective of reason) is acceptable before it is classified as treatment extension. c. Twenty-one days or fewer of non-study anti-TB medications given for treatment against active TB.  b) Re-start of treatment for active TB;  c) Disconutinuation of more than one drug in a specific regimen (excluding frequency or dosage adjustment of one drug) for any reason except reinfection, pregnancy, or temporary drug challenge. |
| **Favorable outcome** | A participant will be classified as having a favorable outcome if any one of the following conditions is met and an unfavorable outcome has not occurred:  • Participants whose last culture result during the Month 9 analysis visit window is MTB Negative and there is no positive culture on visits thereafter till the end of follow-up.  • Participants who are seen during the Month 9 analysis visit window and are clinically without symptoms/signs of ongoing active TB (indicated by absence of initiation of possible poor treatment response evaluation or absense of symptoms/signs of ongoing active TB), and have achieved culture conversion prior to the Month 9 (for the 9-month regimen), and there is no positive culture on visits thereafter till the end of follow-up, and a) Are unable to produce a sputum specimen at any point during the Month 9 analysis visit window; or b) Produce a sputum specimen that is contaminated or unevaluable without evidence of MTB, and no sputum specimens yield positive or negative culture results during the Month 9 analysis visit window. |
| **Not assessable** | A participant will be classified as having a Not Assessable outcome if any one of the following conditions is met and an unfavorable outcome has not occurred:  • Participants not otherwise classified as unfavorable, but do not attend a visit within the Month 21 analysis visit window and thereafter, and their last culture result is negative for MTB.  • Women who become pregnant during assigned study treatment and subsequently be excluded after enrollment.  • Participants who die during the follow-up phase of any cause that is not considered related to tuberculosis.  • Participants who die from a violent (e.g. homicide) or accidental (e.g. road traffic) cause during their assigned study treatment. As above, suicide will be considered an unfavorable outcome.  • Participants who are:  a) Retreated, or have treatment changed or extended; and  b) Demonstrated to be re-infected with a strain of *MTB*, different from that or those identified at study entry through genotyping methods. |

Each participant will be classified into one of the following three outcome categories: 1. Absence of Cure (Unfavorable Outcome) 2. Cure (Favorable Outcome), or 3. Not assessable.

The primary outcome is defined as 21 months after study treatment assignment. Actual visit dates, rather than scheduled visit dates (e.g. Month 2, or Month 9), will be used for all analyses. Only data up to the end of the Month 21 analysis visit window will be included in the primary analysis of the primary efficacy outcome.
