# Supplementary material for: Study protocol for safety and efficacy of all-oral shortened regimens for multidrug-resistant tuberculosis: a multicenter randomized withdrawal trial and a single-arm trial [SEAL-MDR]
Source: BMC Infect Dis. 2023 Nov 27;23:834. doi: 10.1186/s12879-023-08644-8 (PMC10683225; doi:10.1186/s12879-023-08644-8)
Supplement: Supplementary file 2 — Supplementary Material 2 [file 12879_2023_8644_MOESM2_ESM.docx]

**Table S3 Study timeline for 6-month arm**

| **Visit window** |  | **Up to 7 days after screen** | **± 3 days** | | | | **±7 days** | | | | **±7 days** | | | | |  |  |
| --- | --- | --- | --- | --- | --- | --- | --- | --- | --- | --- | --- | --- | --- | --- | --- | --- | --- |
| **Visit** | **Screen** | **Baseline** | M 0.5 | **M 1** | **M 1.5** | **M 2** | **M 3** | **M 4** | **M 5** | **M 6** | **M9** | **M 12** | **M 15** | **M 18** | **M 21** | **Possible poor treatment response** | **Post early termination visit** |
| **Informed consent** | X |  |  |  |  |  |  |  |  |  |  |  |  |  |  |  |  |
| **Inclusion/Exclusion** | X | X |  |  |  |  |  |  |  |  |  |  |  |  |  |  |  |
| **Demographics, medical history** | X |  |  |  |  |  |  |  |  |  |  |  |  |  |  |  |  |
| **Contact information** | X | X | X | X | X | X | X | X | X | X | X | X | X | X | X | X | X |
| **Interval medical history** |  |  |  |  |  |  |  |  |  |  | X | X | X | X | X | X |  |
| **Symptoms** |  | X | X | X | X | X | X | X | X | X | X | X | X | X | X | X | X |
| **Concomitant medications** |  | X | X | X | X | X | X | X | X | X | X | X | X | X | X | X | X |
| **Height** | X |  |  |  |  |  |  |  |  |  |  |  |  |  |  |  |  |
| **Weight (kg)** | X | X | X | X | X | X | X | X | X | X | X | X | X | X | X | X | X |
| **Visual tests** |  | X |  |  |  | X |  | X |  | X |  |  |  |  |  |  |  |
| **Michigan Neuropathy Screening Instruments, MNSI** | X | X | X | X | X | X | X | X | X | X | X | X | X | X | X |  |  |
| **Self-rating depression scale, Self-rating Anxiety Scale** | X |  |  | X |  | X |  | X |  | X |  |  |  |  |  |  |  |
| **HIV test** | X |  |  |  |  |  |  |  |  |  |  |  |  |  |  |  |  |
| **Pregnancy testing (urine or blood)** | X |  |  |  |  |  |  |  |  |  |  |  |  |  |  |  |  |
| **Diabetes screen** | X |  |  |  |  |  |  |  |  |  |  |  |  |  |  |  |  |
| **non-random assignment** |  | X |  |  |  |  |  |  |  |  |  |  |  |  |  |  |  |
| **Sputum for smear and culturec** | X | X | X | X | X | X | X | X | X | X | X | X | X | X | X | X |  |
| **Sputum for rapid molecular test, if available at site** | X |  |  |  |  |  |  |  |  |  |  |  |  |  |  |  |  |
| **Phenotypic DST** |  | X |  |  |  |  |  |  |  |  |  |  |  |  |  |  |  |
| **Storage of Mtb bacterial isolate** | X | X | X | X | X | X | X | X | X | X | X | X | X | X | X | X |  |
| **Blood tests (ALT, bilirubin, Serum albumin, creatinine, potassium, hemoglobin, WBC with differential, platelets)** |  | X | X | X | X | X | X | X | X | X | X | X | X | X | X |  | X |
| **Storage of blood,urine,excrement and breath** |  | X | X | X | X | X | X | X | X | X | X | X | X | X | X |  |  |
| **Urine routine test** |  | X |  | X |  | X |  | X |  | X | X | X | X | X | X |  |  |
| **Chest radiograph (CT)** |  | X |  |  |  | X |  | X |  | X | X | X | X | X | X | X | X |
| **Electrocardiogram** | X |  | X | X | X | X |  | X |  | X |  |  |  |  |  |  |  |
| **Liver ultrasound** | X |  |  |  |  |  |  |  |  |  |  |  |  |  |  |  |  |
| **Adverse events** |  | X | X | X | X | X | X | X | X | X | X | X | X | X | X |  | X |
| **Contact central study clinician** |  | X |  |  |  |  |  |  |  | X |  |  |  |  |  | X |  |

**Table S4 Study timeline for 9-month arm**

| **Visit window** |  | **Up to 7 days after screen** | **± three (3) days** | | | | **±seven (7) days** | | | | | | | **±seven (7) days** | | | |  |  |
| --- | --- | --- | --- | --- | --- | --- | --- | --- | --- | --- | --- | --- | --- | --- | --- | --- | --- | --- | --- |
| **Visit** | **Screen** | **Baseline** | M 0.5 | **M 1** | **M 1.5** | **M 2** | **M 3** | **M 4** | **M 5** | **M 6** | **M 7** | **M 8** | **M 9** | **M 12** | **M 15** | **M 18** | **M 21** | **Possible poor treatment response** | **Post early termination visit** |
| **Informed consent** | X |  |  |  |  |  |  |  |  |  |  |  |  |  |  |  |  |  |  |
| **Inclusion/Exclusion** | X | X |  |  |  |  |  |  |  |  |  |  |  |  |  |  |  |  |  |
| **Demographics, medical history** | X |  |  |  |  |  |  |  |  |  |  |  |  |  |  |  |  |  |  |
| **Contact information** | X | X | X | X | X | X | X | X | X | X | X | X | X | X | X | X | X | X | X |
| **Interval medical history** |  |  |  |  |  |  |  |  |  |  |  |  |  | X | X | X | X | X |  |
| **Symptoms** |  | X | X | X | X | X | X | X | X | X | X | X | X | X | X | X | X | X | X |
| **Concomitant medications** |  | X | X | X | X | X | X | X | X | X | X | X | X | X | X | X | X | X | X |
| **Height** | X |  |  |  |  |  |  |  |  |  |  |  |  |  |  |  |  |  |  |
| **Weight (kg)** | X | X | X | X | X | X | X | X | X | X | X | X | X | X | X | X | X | X | X |
| **Visual tests** |  | X |  |  |  | X |  | X |  | X |  |  | X |  |  |  |  |  |  |
| **Michigan Neuropathy Screening Instruments, MNSI** | X | X | X | X | X | X | X | X | X | X | X | X | X | X | X | X | X |  |  |
| **Self-rating depression scale, Self-rating Anxiety Scale** | X |  |  | X |  | X |  | X |  | X |  |  | X |  |  |  |  |  |  |
| **HIV test** | X |  |  |  |  |  |  |  |  |  |  |  |  |  |  |  |  |  |  |
| **Pregnancy testing (urine or blood)** | X |  |  |  |  |  |  |  |  |  |  |  |  |  |  |  |  |  |  |
| **Diabetes screen** | X |  |  |  |  |  |  |  |  |  |  |  |  |  |  |  |  |  |  |
| **non-random assignment** |  | X |  |  |  |  |  |  |  |  |  |  |  |  |  |  |  |  |  |
| **Sputum for smear and culturec** | X | X | X | X | X | X | X | X | X | X | X | X | X | X | X | X | X | X |  |
| **Sputum for rapid molecular test, if available at site** | X |  |  |  |  |  |  |  |  |  |  |  |  |  |  |  |  |  |  |
| **Phenotypic DST** |  | X |  |  |  |  |  |  |  |  |  |  |  |  |  |  |  |  |  |
| **Storage of Mtb bacterial isolate** | X | X | X | X | X | X | X | X | X | X | X | X | X | X | X | X | X | X |  |
| **Blood tests (ALT, bilirubin, Serum albumin, creatinine, potassium, hemoglobin, WBC with differential, platelets)** |  | X | X | X | X | X | X | X | X | X | X | X | X | X | X | X | X |  | X |
| **Storage of blood,urine,excrement and breath** |  | X | X | X | X | X | X | X | X | X | X | X | X | X | X | X | X |  |  |
| **Urine routine test** |  | X |  | X |  | X |  | X |  | X |  |  | X | X | X | X | X |  |  |
| **Chest radiograph (CT)** |  | X |  |  |  | X |  | X |  | X |  |  | X | X | X | X | X | X | X |
| **Electrocardiogram** | X |  | X | X | X | X |  | X |  | X |  |  | X |  |  |  |  |  |  |
| **Liver ultrasound** | X |  |  |  |  |  |  |  |  |  |  |  |  |  |  |  |  |  |  |
| **Adverse events** |  | X | X | X | X | X | X | X | X | X | X | X | X | X | X | X | X |  | X |
| **Contact central study clinician** |  | X |  |  |  |  |  |  |  | X |  |  | X |  |  |  |  | X |  |
