# Supplementary material for: Study protocol for safety and efficacy of all-oral shortened regimens for multidrug-resistant tuberculosis: a multicenter randomized withdrawal trial and a single-arm trial [SEAL-MDR]
Source: BMC Infect Dis. 2023 Nov 27;23:834. doi: 10.1186/s12879-023-08644-8 (PMC10683225; doi:10.1186/s12879-023-08644-8)
Supplement: Supplementary file 3 — Supplementary Material 3 [file 12879_2023_8644_MOESM3_ESM.docx]

**Table S5 Management of adverse drug reactions and treatment interruptions in the SEAL-MDR study**

| **Adverse reactions** | **Possible Responsible Drugs** | **Management** |
| --- | --- | --- |
| Peripheral neuritis (tremor, numbness, pain in extremities, etc.) | Possible responsible drugs: linezolid, cycloserine, isoniazid, fluoroquinolones, prothionamide, ethambutol.  Drugs focused on in this study: linezolid. | 1. assess the severity of neuritis using scales (Michigan Neuropathy Screening Scale, Short Form McGill Pain Questionnaire), EMG.  2. Depending on the degree of severity, the following measures are selected in order:  (1) Addition of nutritive neurologic drugs. Vitamin B6 (50mg for adults, 25mg for children, <12.5mg for children <5 years old, orally once daily). Methylcobalamin (500ug each time, orally 3 times daily).  (2) Consider using pregabalin [pain reliever, anti-anxiety] or gabapentin [pain reliever, numbness reliever] (non-opioid) to treat pain. Other pain medications such as tramadol (weak opioid) may also be used. Avoid tricyclic antidepressants, which can cause prolongation of the QT interval.  (3) Acupuncture for pain for 2-3 weeks (if available).  (4) Halve the dose of linezolid (300 mg once daily).  (5) Discontinuation of linezolid (needs to be reported to and discussed with the Quality Control Group).  [Note that discontinuation for ≤ 2 weeks is not considered a medication change if use can be resumed. Discontinuation for ≥2 weeks is considered a medication change. The same applies below.] |
| Loss of vision or optic neuritis | Possible responsible drugs: linezolid, ethambutol, prothionamide.  Drugs focused on in this study: linezolid. | 1. If you cannot distinguish the responsible drug, consider discontinuing prothionamide, ethambutol, and linezolid in that order for observation. Gradually stop the drug and observe the recovery of vision. If vision continues to decline despite stopping a drug for 2 weeks, consider other responsible drugs as soon as possible.  2. If ethambutol is considered the responsible drug, choose the following measures in order:  (1) Monitor changes in vision and seek ophthalmologic consultation.  (2) Addition of nutritive neurologic drugs. Vitamin B6 (50mg for adults, 25mg for children, <12.5mg for children <5 years old, orally once daily). Methylcobalamin (500ug each time, 3 times daily).  (3) Discontinue ethambutol, report to the Quality Control Group and discuss.  (4) Those with concomitant diabetes should have better control of their blood sugar levels.  3. If linezolid is considered the responsible drug, choose the following measures in order:  (1) Addition of nutritive neurologic drugs. Vitamin B6 (50mg for adults, 25mg for children, <12.5mg for children <5 years old, orally once daily). Methylcobalamin (500ug per dose, orally 3 times daily).  (2) If vision loss is significant in the short term, linezolid needs to be discontinued immediately or permanent visual impairment may result.  (3) Halve the dose of linezolid (300 mg once daily).  (4) Discontinuation of linezolid (needs to be reported and discussed with the Quality Control Group). Discontinuation of ≤ 2 weeks is not counted as a medication change if the use can be resumed. Discontinuation for ≥ 2 weeks is counted as a medication change.  (5) If the patient has extensive pulmonary lesions or further acquired resistance, reintroduction of linezolid may be considered and carefully monitored after the ophthalmologist has ruled out linezolid-induced visual toxicity when linezolid is considered to be of greater therapeutic significance.  (6) Be alert for the development of drug-induced color blindness (green color blindness is usually the first to appear) and consult an ophthalmologist if necessary. |
| Bone marrow suppression (anemia, thrombocytopenia, leukopenia) | Possible responsible drugs: linezolid, fluoroquinolones, rifamycins, isoniazid.  Drugs focused on in this study: linezolid, fluoroquinolones. | 1. Before treatment:  (1) In the case of moderate to severe anemia (Hb < 80 g/L), the underlying disease needs to be checked and treated.  (2) Initiating linezolid at Hb < 80 g/L would be considered only if the patient is closely monitored in the hospital and transfusion is an option. If close monitoring and/or hospitalization is not possible, linezolid should be avoided.  (3) If linezolid is started in the hospital but hemoglobin does not improve and stabilizes above 80 g/L, other drugs need to be substituted for linezolid.  2. During treatment:  (1) For mild to moderate adverse reactions, close monitoring or use of symptomatic management drugs, such as platelet and white blood cell elevating drugs.  (2) When hemoglobin drops to <80g/L, platelets <50*109L, and leukocytes <1*10^9^L, sequentially stop linezolid and fluoroquinolones and observe any improvement, investigate other possible causes, and recommend hospitalization. In case of severe anemia, thrombocytopenia and leukopenia, transfusion of red blood cell suspension, single platelet collection and granulocyte colony-stimulating factor can be used.  (3) If myelosuppression resolves and linezolid is important to this regimen, consider reintroducing linezolid at a low dose (300 mg/d or 600 mg/three times per week, in a single dose), but with increased monitoring; intermittent use of linezolid may also be considered if myelosuppression recurs after resumption of linezolid.  (4) If myelosuppression resolves and fluoroquinolones are important to the regimen, reintroduction of fluoroquinolones (without dose reduction) may be considered, but intensive monitoring is required. |
| QTcF prolongation | Possible responsible drugs: bedaquiline, clofazimine, delamanid, fluoroquinolones, clarithromycin, azithromycin.  Drugs focused on in this study: bedaquiline, clofazimine, fluoroquinolones. | When it is not possible to distinguish the responsible drug, it is recommended that clofazimine, fluoroquinolones, and bedaquiline be discontinued in that order.  1. Baseline: QTcF >450ms, then bedaquiline is not indicated; caution should be exercised with other potentially hazardous medications such as fluoroquinolones, clofazimine, etc. The frequency of QTcF monitoring for ECGs is set at 2 weeks, 4 weeks, and monthly (for the first 6 months) at the start of treatment. The frequency of monitoring can be increased according to the situation.  2. Treatment period:  (1) QTcF <450 ms, continue with bedaquiline.  (2) QTcF ≥450 ms and <469 ms is considered to be a mild prolongation of QTcF, and ECG continues to be monitored once a month.  (3) QTcF ≥470 ms and <499 ms were considered moderately prolonged. a) If there were no cardiac clinical symptoms (chest pain, palpitations, vertigo, syncope), bedaquiline therapy was continued, and ECG monitoring was repeated in 1 week. b) If the clinical symptoms described above were present, bedaquiline and other suspected medications were discontinued, and ECGs were repeated in 1 week.  (4) QTcF ≥500ms, or its increase ≥50ms, is considered to be severely prolonged.  a) Discontinue bedaquiline, fluoroquinolones, and clofazimine immediately.  b) Examine other risk factors for prolongation of the QT interval (other medications, electrolyte disorders, and hypothyroidism) and treat them appropriately.  c) Check blood potassium, calcium, and magnesium levels and recommend maintaining blood potassium levels above 4mmol/L. Check blood potassium, calcium and magnesium levels, and it is recommended to keep the blood potassium level higher than 4 mmol/L and the blood magnesium level higher than 0.74 mmol/L. Concomitant use of medications that may prolong the QTc interval should be avoided, e.g., cisapride, erythromycin, antipsychotics and tricyclic antidepressants, etc.  d) Review the ECG after 48 hours, and change to weekly monitoring of the ECG if the QTcF decreases.  e) If the QTcF is lower than 470 ms, sequentially repeat the ECG after 48 hours.  f) If the ECG is lower than 470 ms, repeat the ECG after 48 hours. 470ms, sequentially restart fluoroquinolones (it is recommended to replace moxifloxacin with high-dose levofloxacin), clofazimine, and bedaquiline, and monitor ECG during this period.If QTcF is still >470ms within 2 weeks, cardiology consultation should be requested for management. |
| Arthralgia or myalgia | Possible responsible drugs: pyrazinamide, fluoroquinolones, linezolid, bedaquiline.  Drugs focused on in this study: pyrazinamide, fluoroquinolones, linezolid. | 1. If it is difficult to differentiate between the responsible drugs, discontinue pyrazinamide and fluoroquinolones in sequence and observe. Note that in some cases the symptoms of arthralgia will gradually resolve with prolonged treatment, even if no intervention is given.  2. If pyrazinamide is considered to be the responsible drug, the following measures may be selected in sequence:  (1) Monitor blood uric acid. If no joint pain occurs, hyperuricemia may be left untreated.  (2) If there is joint pain, the following measures may be selected in order:  a) Drink plenty of fluids, eat less high uric acid foods, reduce joint activity, and observe pain changes.  b) Start symptomatic treatment with NSAIDs. Example: Ibuprofen 600mg three times a day.  c) Pyrazinamide reduce the dose to 1.0 g once daily.  d) Pyrazinamide discontinuation (to be reported to and discussed with the Quality Control Group).  e) If there is severe swelling of the joints, reddening of the skin, and elevated skin temperature, consider a puncture biopsy to rule out gout, infection, or auto immune disease. If a gouty attack occurs and is considered drug-induced, discontinue pyrazinamide immediately.  3. If a fluoroquinolone is considered the responsible drug, the following measures may be selected in sequence:  a) Consider using pregabalin or gabapentin to treat pain.  b) Focus on Achilles tendon pain and may request an orthopedic consultation.  c) If there is a short-term noticeable pain aggravation or even affecting walking, the fluoroquinolones need to be stopped immediately.  4. If linezolid is considered the responsible drug, refer to linezolid side effect management. |
| Achilles tendonitis | Fluoroquinolones | 1. Start symptomatic treatment with NSAIDs. Example: Ibuprofen 600mg three times a day.  2. Reduce joint activity and observe pain changes.  3. If the pain worsens significantly in a short period of time or even affects walking, the fluoroquinolones should be stopped immediately. |
| Hyperuricemia | Possible responsible drugs: pyrazinamide, ethambutol.  Drugs focused on in this study: pyrazinamide. | Refer to Pyrazinamide-induced joint pain management. |
| Gastrointestinal symptoms, nausea, vomiting, etc. | Possible responsible drugs: prothionamide, para-aminosalicylic acid, isoniazid, ethambutol, pyrazinamide, clofazimine, bedaquiline.  Drugs focused on in this study: prothionamide, pyrazinamide. | 1. If you cannot differentiate between the responsible drugs, please consider prothionamide and pyrazinamide in that order.  a) Evaluate the severity of the symptoms. If the symptoms are mild or moderate, they can be treated symptomatically without stopping the drug. If the mild or moderate symptoms do not improve after treatment and the symptoms gradually worsen and severe symptoms appear, stop the suspected drug and observe the improvement of the symptoms after stopping the drug.  b) New onset nausea and vomiting requires consideration of hepatotoxicity, hepatitis, pancreatitis, elevated intracranial pressure, or pregnancy. If the patient's vomit is bloody, hemoglobin indices should be tested and possible gastrointestinal bleeding disorders should be treated.  2. The following measures may be selected in turn:  (1) Adjust the timing of the medication without reducing the dose. Bedaquiline and clofazimine need to be taken with a meal; other drugs are optional:  a) Take it 2 hours after breakfast.  b) Take 2 hours after dinner. Sometimes, adjusting most of the daytime medication to be taken in the evening may lessen the symptoms of adverse effects.  c) Sometimes a small snack (light meal: crackers, bread, rice, tea, etc.) may be eaten before the medication and may lessen the symptoms of adverse reactions.  (2) Add gastric medication. Metoclopramide 10-20mg 30min before taking anti-tuberculosis drugs. If metoclopramide is not effective, ondansetron 2-8mg should be used 30min before taking anti-tuberculosis drugs.  (3) Reduce the dose of the drug. For patients who are overly concerned about the possibility of nausea, give diazepam 5 mg 30 min before dosing.  (4) Discontinuation of suspected drugs.  (5) Be alert for postvomiting hypokalemia. |
| gastritis | Possible responsible drugs: prothionamide, p-aminosalicylic acid.  Drugs focused on in this study: prothionamide. | 1. A light meal before medication is recommended.  2. Note: Cation-containing drugs can reduce the absorption of fluoroquinolones, such as magnesium and aluminum (and thioclates) (highly reducing); iron (moderately reducing); and calcium and zinc (and multivitamins) (low reducing).  3. Omeprazole 20-40 mg given at night (2 hours before or 3 hours after dosing). |
| Diarrhea | Possible responsible drugs: linezolid, para-aminosalicylic acid, prothionamide, fluoroquinolones, clofazimine.  Drugs focused on in this study: linezolid. | 1. For mild diarrhea, encourage the patient to tolerate it.  2. Encourage rehydration therapy.  3. If diarrhea is present but there is no blood in the stool, fever, etc., take loperamide 4mg if necessary, then 2mg after each bowel movement up to a maximum of 10mg in 24 hours. |
| Lactic acidosis (symptoms: abdominal pain, nausea, vomiting, deep shortness of breath, generalized weakness) | Linezolid | Lactic acidosis can be monitored by blood tests for lactate levels. If lactic acidosis occurs, discontinue linezolid. |
| Dizziness/floating sensation when walking | Possible responsible drugs: cycloserine, fluoroquinolones, clofazimine, isoniazid.  Drugs focused on in this study: cycloserine. | Cycloserine is the primary drug responsible for dizziness, please select the following measures in order:  1. Vitamin B6 prevents the neurotoxic effects of cycloserine, and the recommended dose is 50 mg of vitamin B6 orally per 250 mg of cycloserine. It is not recommended to increase the dose of vitamin B6 to >100 mg to minimize the neurotoxicity inherent in prolonged use of high doses of vitamin B6 (e.g., 200 mg once daily for >1 month).  2. Assess the severity of dizziness and advise the patient not to drive or perform other hazardous maneuvers that require a sense of balance during dizziness.  3. Adjust the time of medication. If the medication was originally taken once in the morning and once in the evening, consider changing it to once in the evening, which may reduce the inconvenience caused by dizziness during the day.  4. Reduce cycloserine dose to 25mg-50mg daily.  5. If cycloserine is discontinued, this needs to be reported and discussed with the Quality Control Group.  6. Focus on observing the psychotoxic side effects caused by cycloserine, such as severe insomnia, depression. Anxiety, suicidal tendency, etc. Cycloserine needs to be discontinued immediately if present. To minimize serious adverse events such as suicide, family members of the patient are advised to closely monitor the patient's mental changes, or healthcare professionals and the patient should maintain close contact. |
| Headache | Possible responsible drugs: cycloserine, bedaquiline, linezolid, fluoroquinolones.  Drugs focused on in this study: cycloserine. | 1. Rule out more serious causes of headache, including meningitis and other central nervous system infections (undergo a CT scan of the head and cerebrospinal fluid analysis if necessary).  2. Mild headaches are treated with analgesics such as ibuprofen or acetaminophen.  3. Tricyclic antidepressants were once the drugs of choice, but their clinical use has been limited by their high anticholinergic and cardiovascular adverse effects, numerous contraindications and drug interactions, and narrow safety range. Psychiatric drugs and tricyclic antidepressants (promethazine, clomipramine, doxepin hydrochloride and amitriptyline) that can cause QT interval prolongation should be avoided in this study. |
| Seizures | Possible responsible drugs: cycloserine, isoniazid, fluoroquinolones.  Drugs focused on in this study: cycloserine, moxifloxacin. | 1. Vigilantly check creatinine levels in patients with sudden seizures. Renal impairment can lead to elevated serum cycloserine concentrations.  2. Start anticonvulsant therapy (carbamazepine, phenytoin or valproic acid).  3. Replace cycloserine with other drugs if necessary. |
| Memory loss | Cycloserine | 1. Usually mild, well tolerated, and reversible on discontinuation of the drug.  2. Be aware of other side effects of cycloserine. |
| Fatigue | cycloserine (Cycloserine), an essential amino acid | 1. Usually mild, well tolerated, and reversible on discontinuation of the drug.  2. Be aware of other side effects of cycloserine. |
| Mental illness (depression, anxiety) | Possible responsible medications or conditions: cycloserine, fluoroquinolones, high-dose isoniazid, prothionamide, etc.; underlying psychological disorders; socioeconomic factors.  Drugs focused on in this study: cycloserine, fluoroquinolones. | When it is not possible to differentiate between the responsible drugs, it is recommended that prothionamide, cycloserine, and fluoroquinolones be discontinued sequentially. Try to consider the patient's socioeconomic issues into the program development process and choose the appropriate treatment plan.  When considering cycloserine as the responsible drug, the following measures were selected in order:  1. Maintain close and open contact with the patient to detect suspected psychosomatic changes at an early stage. 2. Conduct regular cardiac evaluations, use depression and anxiety scales, and seek psychosocial consultation when necessary.  3. Cycloserine may still be considered for patients with underlying psychiatric psychiatric disorders who have been evaluated and effectively monitored, and for whom there is no other effective drug alternative to cycloserine. Do not discontinue the original psychiatric-psychological specialty drugs, but be alert: (1) Fluoxetine, amitriptyline, may induce serotonin syndrome when used in combination with linezolid. (2) Amitriptyline, by itself, can cause QT interval prolongation, try not to use with bedaquiline, fluoroquinolones, clofazimine.  4. Cycloserine should be avoided if depressive symptoms are evident at the beginning of treatment, and monitoring of blood levels is recommended when conditions permit.  5. If necessary, reduce the dose of the drug to 500 mg per day of cycloserine and prothionamide.  6. Always be alert to creatinine levels in patients with sudden-onset depression, as impaired renal function can increase cycloserine serum concentrations. |
| Skin pigmentation | Clofazimine | 1. Record the skin changes before and after the use of clofazimine and take photographs.  2. Inform the patient that the skin changes can be gradually restored to near normal after stopping the drug for six months to two years, and give psychological comfort.  3. Patients are advised to reduce sun exposure, drink plenty of water, and use skin care products; these measures can reduce the degree of skin discoloration.  4. The occurrence of changes in skin color is not an indication for discontinuation, but if the skin changes lead to serious psychological disturbances in the patient, it is necessary to consult promptly on whether to discontinue clofazimine.  5. In addition to skin discoloration, for dry, cracked, ichthyosis-like changes in the skin, dermatology consultation and symptomatic treatment can be requested. |
| Liver damage (symptoms: nausea, vomiting, abdominal pain, jaundice.) | Possible responsible drugs: (1) greater hepatic damage: pyrazinamide; (2) possible hepatic damage: prothionamide, isoniazid, para-aminosalicylic acid, clofazimine, bedaquiline. (3) Little to no liver damage: linezolid, delamanid, fluoroquinolones.  Drugs focused on in this study: pyrazinamide, prothionamide. | 1. The patient's tuberculosis condition, degree of liver injury, associated risk factors and general condition should be comprehensively assessed before treatment.  2. Only alanine aminotransferase ALT <3 times the upper limit of normal value (ULN) without obvious symptoms and jaundice can be treated with liver-protecting therapy under close observation and discontinuation of anti-tuberculosis drugs with a high frequency of liver injury, as appropriate.  3. If ALT ≥ 3 times ULN or total bilirubin ≥ 2 ULN, discontinue anti-tuberculosis drugs with a high frequency of liver injury, and perform hepatoprotective therapy with close observation;  4. ALT ≥ 5 times ULN or ALT ≥ 3 times ULN with symptoms such as jaundice, nausea, vomiting, malaise, etc., or total bilirubin ≥ 3 ULN, all anti-tuberculosis drugs should be immediately discontinued, and liver-protecting treatment should be actively carried out; patients with severe liver injury should be hospitalized to take comprehensive treatment measures, and resuscitation measures should be actively taken in case of manifestations of liver failure.  5. wait for liver function to return to normal or at least <3 ULNs. drugs should be reintroduced sequentially every 5-7 days and liver function monitored before introducing the next drug. The least hepatotoxic drug (linezolid, fluoroquinolones) should be added first, and then potentially hepatotoxic drugs (clofazimine, bedaquiline) should be introduced one at a time every 5 to 7 days while monitoring liver function tests to identify the drug of interest. Pyrazinamide should not normally be reintroduced.  6. If reintroduction of a drug results in recurrence of symptoms of liver injury and elevation of hepatic aminotransferases, permanent discontinuation of the drug should be considered. |
| Kidney injury | Possible responsible drugs: kanamycin, amikacin, colistin, ethambutol, pyrazinamide, cycloserine. | If necessary, change ethambutol, pyrazinamide to three times weekly. |
| Thyroid Diseases | Prothionamide | 1. Generally reversible after discontinuation of the drug.  2. If thyroid-stimulating hormone (TSH) levels are elevated, evaluate for symptoms of hypothyroidism.  3. If TSH > 1.5-2 times the upper limit of normal, start specialty treatment.  4. Levothyroxine 100-150 µg/day for adults, 75-100 µg/day for adolescents; 50 µg/day for the elderly (>65 y); 25 µg for severe cardiovascular disease.  5. Reassess TSH levels after 1-2 months and adjust levothyroxine dose accordingly. |
| Rash, allergic reaction | Any drug | 1. Mild reactions usually disappear on their own a few weeks after the start of treatment, and it is not necessary to stop the medication. Antihistamines, such as cetirizine 5-10mg or diphenhydramine 25-50mg, may also be used before taking anti-tuberculosis drugs. If skin irritation is more pronounced, a hormone-containing skin cream may be applied. If the skin is dry, use sunscreen and moisturizer.  2. If none of the above treatments are effective, low-dose oral hormones, such as prednisolone 10-20 mg daily for several weeks, may be given.  3. In severe anaphylactic reactions, where the rash is generalized, accompanied by systemic symptoms such as fever, abnormalities of visceral function, and in severe cases, involvement of mucous membranes, as well as the production of loose skin flaking, all therapeutic medications should be discontinued immediately, and the allergic reaction should be managed using standard emergency protocols until the allergic state improves.  4. Wait for the patient's allergic status to completely return to normal (it may take 1 week or longer), and then try the therapeutic drugs one by one, starting with the drugs that are the least likely to cause allergic reactions, and not recommending the re-use of highly suspected allergic drugs in principle.  5. Patients should be carefully asked about their previous drug allergy history, and drugs with a clear history of allergy should be identified on the patient's treatment card and not selected, and attention should be paid to avoiding the use of drugs with the possibility of cross-allergic reactions.  6. Skin discoloration caused by clofazimine is not an allergic reaction, but needs to be monitored for mental health effects.  7. Rule out other potential causes of allergic skin reactions (e.g., scabies or other environmental factors). |
| Dysglycemia | Prothionamide | 1. Usually mild, well tolerated, and reversible on discontinuation of the drug.  2. Be aware of other side effects of prothionamide. |
| Lose hair or feathers | Prothionamide | 1. Usually mild, well tolerated, and reversible on discontinuation of the drug.  2. Be aware of other side effects of prothionamide. |
| Black hairy tongue | A rare side effect of linezolid. | Black hairy tongue may subside when the dose of linezolid is reduced or discontinued. However, no other serious side effects of black hairy tongue have been reported, so reduction or discontinuation of treatment is not usually necessary. |

Most of the following adverse events (AEs) are reversible, do not cause permanent damage or dysfunction, and need to be communicated in detail when explaining the condition to the patient. However, serious adverse events (SAEs) are potentially life-threatening or may cause permanent damage (grades 3 and 4) and should be managed by an experienced clinician or a panel of specialists in order to identify the suspected medication, reduce the dose, or discontinue use; discontinuation of the medication is the final option only after adjunctive management measures have been used in sequence and have failed.

**Table S6 Management of treatment interruptions in the SEAL-MDR study**

| **Duration of treatment interruptions** | **Treatment period^*^** | **Is it necessary to do**  **sputum examination** | **Sputum results** | **Management** |
| --- | --- | --- | --- | --- |
| <14 days | - | No | - | Continue the original regimen with extended treatment period to make up for the interruption |
| ≥14 days, <2 months | Intensive period (2 months before treatment) | Yes | Negative | Restart treatment^**^ |
|  |  |  | Positive | Carry out drug susceptibility tests; Restart treatment |
|  | Continuation period (2 months of treatment - end of course) | Yes | Negatives | Continue the original regimen with extended treatment period to make up for the interruption |
|  |  |  | Positive | Carry out drug susceptibility tests; continue the original regimen with extended treatment period to make up for the interruption |

*Categorized by interruption of treatment occurring during the intensive and continuation phases of treatment. If it straddles the intensification and continuation periods, it is determined by the fact that the interruption of treatment occurred during the intensification period.

**That is, the treatment program is restarted and previous treatments are not counted.

For patients with consecutive treatment interruptions (meaning interruptions of any 2 or more antituberculosis drugs) of ≥2 months, they are considered cases of protocol violation and need to be documented in adverse events and need to be withdrawn early from this study. For patients with less than 2 months of continuous treatment interruption (meaning interruption of any two or more anti-tuberculosis drugs), they need to be recorded in adverse events, and the appropriate treatment regimen needs to be selected based on the patient's duration of treatment interruption, the period of treatment they are on, and their sputum results, among other things, which should be assessed.
