# Supplementary material for: Study protocol for safety and efficacy of all-oral shortened regimens for multidrug-resistant tuberculosis: a multicenter randomized withdrawal trial and a single-arm trial [SEAL-MDR]
Source: BMC Infect Dis. 2023 Nov 27;23:834. doi: 10.1186/s12879-023-08644-8 (PMC10683225; doi:10.1186/s12879-023-08644-8)
Supplement: Supplementary file 4 — Supplementary Material 4 [file 12879_2023_8644_MOESM4_ESM.docx]

**S4 Participating Hospitals**

**Table S8** The names of the 32 participating hospitals arranged in alphabetical order based on their English names.

| 01 | Anhui Chest Hospital(Tuberculosis Prevention and Control Institute of Anhui Province) |
| --- | --- |
| 02 | Ankang Central Hospital |
| 03 | Centre for Tuberculosis Control of Guangdong Province |
| 04 | Chest Hospital of Guangxi Zhuang Autonomous Region (The Forth People's Hospital of Guangxi Zhuang Autonomous Region) |
| 05 | Chongqing Public Health Medical Treatment Center（Southwest University Public Health Hospital） |
| 06 | Guangzhou Chest Hospital |
| 07 | Guiyang Public Health Treatment Center |
| 08 | Heilongjiang Provincial Institute for the Prevention and Control of infectious Diseases |
| 09 | Henan Province Chest Hospital |
| 10 | Inner Mongolia Fourth Hospital |
| 11 | Jiangmen Tuberculosis Prevention and Control Institute |
| 12 | Jiangxi Province Chest Hospital |
| 13 | Shenzhen Third People's Hospital |
| 14 | The Eighth Affiliated Hospital of Xinjiang Medical University |
| 15 | The Fifth Affiliated Hospital of Sun Yat-sen University |
| 16 | The Fifth People's Hospital of Ganzhou |
| 17 | The fourth People’s Hospital of Foshan |
| 18 | The Fourth People’s Hospital of Nanning |
| 19 | The Second Affiliated Hospital of Hainan Medical College |
| 20 | The Sixth People's Hospital of Zhengzhou |
| 21 | The Sixth People’s Hospital of Dongguan |
| 22 | The Third People's Hospital of Aksu |
| 23 | The Third People's Hospital of Guiling |
| 24 | The Third People's Hospital of Jiujiang |
| 25 | The Third People's Hospital of Shantou |
| 26 | The Third People's Hospital of Tibet Autonomous Region |
| 27 | The Third People's Hospital of Wuzhou |
| 28 | Tuberculosis Prevention and Control Institute of Hunan Province（Hunan Chest Hospital） |
| 29 | Yangjiang Public Health Hospital |
| 30 | Yingde Chronic Hospital |
| 31 | Yulin Red Cross Hospital |
| 32 | Zigong First People's Hospital |
