# Supplementary material for: Study protocol for safety and efficacy of all-oral shortened regimens for multidrug-resistant tuberculosis: a multicenter randomized withdrawal trial and a single-arm trial [SEAL-MDR]
Source: BMC Infect Dis. 2023 Nov 27;23:834. doi: 10.1186/s12879-023-08644-8 (PMC10683225; doi:10.1186/s12879-023-08644-8)
Supplement: Supplementary file 5 — Supplementary Material 5 [file 12879_2023_8644_MOESM5_ESM.docx]

**Safety and Efficacy of All-oral Shortened Regimens for Multidrug-Resistant Tuberculosis: A Multicenter Randomized Withdrawal Trial and A Single-arm Trial [SEAL-MDR]**

**Informed Consent**

**Participant Initials:**

**Participant number:**

**Dear patient:**

We invite you to participate in a multicenter randomized drug withdrawal trial of an all-oral short-course regimen for the treatment of multidrug-resistant tuberculosis (MDR-TB), led by the National Center for Clinical Research of Infectious Diseases (Shenzhen)/Shenzhen Third People's Hospital (SZ-3PH), with the technical support of Huashan Hospital, Guangdong Provincial Center for Tuberculosis Control (GDCTC), and the School of Medicine of Shenzhen University (SMU). This study will be conducted at the Third People's Hospital of Shenzhen, the Fourth People's Hospital of Foshan, the Sixth People's Hospital of Dongguan, the Third People's Hospital of Shantou, the Chest Hospital of Guangzhou, the Fifth Affiliated Hospital of Sun Yat-sen University, the Public Health Hospital of Yangjiang, the Tuberculosis Prevention and Control Center of Jiangmen, the Chronic Disease Prevention and Control Hospital of Yingde, the Longtan Hospital of Guangxi Zhuang Autonomous Region, the Third People's Hospital of Wuzhou, the Third People's Hospital of Guilin, the Nanning Fourth People's Hospital, Jiangxi Provincial Chest Hospital, Ganzhou Fifth People's Hospital, Jiujiang Third People's Hospital, Guiyang City Public Health Relief Center, Chongqing Public Health Relief Center, Hunan Provincial Chest Hospital, Anhui Provincial Chest Hospital, Henan Provincial Chest Hospital, Heilongjiang Provincial Tuberculosis Prevention and Control Institute, and the Second Affiliated Hospital of Hainan Medical College. It is expected that ≥1128 patients will participate voluntarily. Study period: 01/01/2022 - 12/31/2025. Some of the content covered in this document is in accordance with the requirements of relevant regulations, and in order to protect the rights of patients participating in the study, this document has been reviewed and agreed by the ethics committees of each center. In addition, this consent form may contain language that you do not understand. If you encounter words or information that you do not clearly understand, ask your study doctor or another member of his or her team to explain them to you.

**Why was this study undertaken?**

Tuberculosis is usually an infection of the lungs with Mycobacterium tuberculosis. The vast majority of patients can be cured after about 6 months of standard anti-tuberculosis drug treatment, but if you are infected with drug-resistant bacteria or if you develop a drug-resistant condition due to irregular treatment, etc., your doctor will adjust the medication and prolong the duration of the treatment for you. Because the traditional treatment regimen for multidrug-resistant tuberculosis (MDR-TB) is long, expensive, and involves many types of drugs with many side effects, patients often have difficulty adhering to the regimen and experience poor clinical outcomes, and even become poorer as a result of the disease, there is an urgent need for research into effective, safe, and affordable treatment regimens. Therefore, there is an urgent need to investigate effective, safe and affordable treatment options. Therefore, there is an urgent need to investigate effective, safe and affordable treatment options. The present study is aimed at exploring an all-oral, short-course treatment regimen suitable for China's national conditions for the benefit of the general patient population.

**How was the study conducted?**

When a patient is diagnosed with rifampicin-resistant or multidrug-resistant tuberculosis, the treatment regimen is presented in detail to the patient and his/her family. Based on the treatment regimens recommended by the World Health Organization and taking into account the domestic drug resources and the different situations of drug resistance in patients, we have preferred four regimens. Based on the drug resistance, tolerability and affordability of specific patients, one of the four treatment regimens was selected and informed consent was signed. The study was open and unblinded, which means that each participant could know his or her medication regimen and course of treatment. However, both short-course and conventional regimens carry the risk of relapse and the possibility of re-infection, and we will identify whether it is a relapse or a re-infection by genetic testing for Mycobacterium tuberculosis.

Specifically, this study was divided into three parts. Part I: a randomized withdrawal trial was conducted, targeting regimens A and B for fluoroquinolone-sensitive MDR-TB, with the following regimens: regimen A: bedaquiline/linazolamide/moxifloxacin/circumcision (Bdq/Lzd/Mfx/Cs), and regimen B: linazolamide/moxifloxacin/circumcision/clofazimine/pyrazinamide (Lzd/Mfx/Cs/Cfz/Z), respectively. A regimen was naturally selected at enrollment, efficacy was assessed at 6 months of treatment, and those with good efficacy within the regimen were randomized: the withdrawal group discontinued all medications immediately, and the continuation group completed the remainder of the regimen. The difference in the incidence of adverse tuberculosis outcomes between the two groups was compared after drug withdrawal and follow-up observations until after drug discontinuation. The aim is to validate the efficacy and safety of the 6-month short-course regimen as non-inferior to the 9-month short-course regimen. Part II: A single-arm clinical trial was conducted to target C and D regimens for fluoroquinolone-resistant MDR-TB. The regimens are: regimen C: Bdq/Lzd/Cs/Cfz/Z, and regimen D: Lzd/Cs/Cfz/Z + salazosulfapyridine (SASP). A regimen was selected naturally at enrollment, and efficacy was assessed at 6 months of treatment to determine the need to extend the course of treatment and to observe the incidence of adverse outcomes. Part III: A real-world study to develop an individualized regimen for MDR-TB who cannot be enrolled in part I or part II. Patients are responsible for their own medication costs.

If you are diagnosed with rifampicin-resistant or multidrug-resistant TB and you wish to participate in this study, your doctor will choose one of the four treatment regimens based on your drug resistance and your wishes. You will be evaluated at 6 months of treatment and, if you are doing well, you will be randomized to either the stop-treatment group or the continue-treatment group. The probability of being placed in either group is like the result of a coin flip, and neither you nor your doctor will be able to choose which group you will be placed in. If we assess that your TB is not responding well at 6 months of treatment, you will be put on anti-tuberculosis treatment for 9 to 12 months or longer.

Regardless of which study group you are enrolled in, we will collect sputum and blood specimens at your regular follow-up appointments, and may also collect additional sputum and blood specimens for testing as your condition changes to better adjust your treatment plan. In addition, regular chest CTs will be needed to assess the improvement of your lung lesions. We may also review records of TB clinic visits during your participation in the study if you develop symptoms of TB again after completing treatment.

Regardless of which group of drugs you are being treated with, we will keep watching you for at least 21 months from the start of the study to see if you still have TB or if you have been cured.

Whether or not you participate in this study is entirely up to you, and whether or not you participate will not affect any treatment you would otherwise receive.

**Conditions to be fulfilled to participate in Part 1 or Part 2 of the trial/study?**

1) Voluntary participation in this study and signed informed consent; in the case of minor patients, guardian consent was required;

(2) Aged between 12 and 75, weighing 30kg or above, male or female;

3) Chest CT suggesting the presence of a pulmonary lesion;

4) Patients with rifampicin-resistant tuberculosis diagnosed by sputum, induced sputum, or GeneXpert MTB/RIF on alveolar lavage;

5) Ability to obtain results on resistance of M. tuberculosis to fluoroquinolones;

6) Patients diagnosed with active tuberculosis by comprehensive clinical assessment and in need of anti-tuberculosis treatment.

7) In the case of women of childbearing age, they will need to have a negative urine test for pregnancy and agree to use highly effective contraception (including condoms, ligation, etc.; pharmaceutical contraception is not recommended) for the duration of the study. In the case of breastfeeding women, they need to agree to stop breastfeeding;

8) Have a recognizable address and remain in the area for the duration of the study.

**You will not be allowed to participate in Part I or Part II of the study if you have one of the following conditions:**

1) Have tuberculosis other than pulmonary;

2) Already resistant to the drugs in the regimen, or there is an allergy, or there is a reluctance to use them;

3) Have used bedaquiline or linezolid for more than 1 month;

4) Indicator of ECG QTcF ≥ 450ms;

5) Certain specific blood indicators are very abnormal, such as those for hemoglobin, platelets, kidney function, and liver function; or the overall condition is very serious;

(6) Concurrently having certain specific diseases, such as mental illness, AIDS; or needing to take certain specific drugs for a long period of time due to the presence of other diseases, such as hormones, antipyretic and analgesic drugs, cardiovascular specialty drugs, antidepressant drugs, etc.

7) Alcoholism, drug addiction;

(8) Pregnant or breastfeeding women, or women of childbearing age who are unwilling to use effective contraception;

9) You are currently participating in a clinical trial of another drug;

(10) You suffer from any other condition that, in the judgment of the subject group, makes you unsuitable to participate in this project.

**What should I do in my research?**

(1) You are required to provide true and complete clinically relevant data and inform the researchers of any unexpected situation;

(2) You must not conceal your medical condition and strictly follow your doctor's instructions while undergoing clinical research;

(3) If it is necessary to terminate this clinical study, the researchers need to be informed in advance for data processing;

(4) During the study period, you will be required to strictly follow the doctor's instructions, receive regular visits to the study doctor, take medication, and receive various clinical examinations on a regular basis, including: providing information on symptomatic changes, adverse reactions, and medication combinations; receiving physical examinations; routine blood tests, urine tests, fecal routines, blood biochemistry, coagulation, and immunity tests, etc., so that you can learn about the safety and effectiveness of your treatment regimen, and adjust the regimen if necessary. The treatment program will be adjusted if necessary, so that your disease can be closely monitored and effectively treated. Please contact your study doctor for follow-up visits. Each visit takes about half a day.

5) HIV Testing: Before we can decide whether or not we can include you in this study, we must test you for Human Immunodeficiency Virus (HIV). If you test positive for HIV, you will not be able to participate in this study and should be referred to an HIV specialist for appropriate treatment. If you decide not to be tested for HIV, you cannot participate in this study. We strongly encourage everyone to know their HIV status and to take steps accordingly, both for their own health and to help our country realize the vision of an AIDS-free generation.

6) Retained samples for future research: In addition to the samples collected during the study for routine testing, the remaining samples will be frozen and used for future TB-related research. All frozen samples will only be stored with a coded identification, so please be assured that your privacy will not be compromised. If you do not agree to have your samples stored, you will not be able to participate in this study.

7) Depending on your treatment regimen, the entire study consists of a treatment period (6-12 months) and a follow-up period after stopping the drug (6-12 months, or longer, to see if there is a recurrence of the disease, etc.).

8) The overall study flow is shown in the table below (for a 6-month course of treatment):


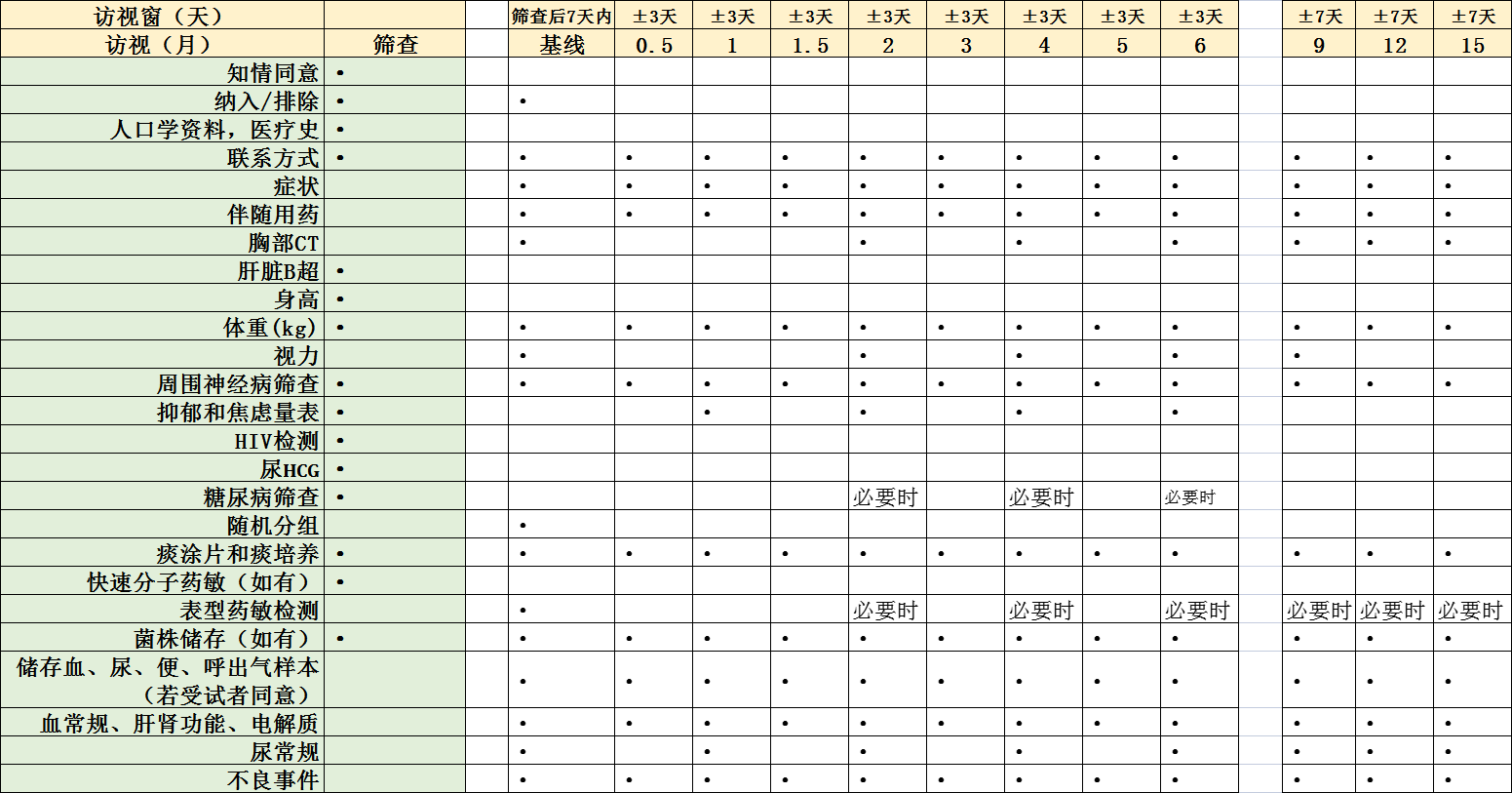


**Do I have other treatment options?**

Participating in this study may or may not improve your health; you have a choice:

 - Do not participate in this study and continue your regular treatment;

 -Participate in another study;

 - not receiving any treatment.

Consult with your doctor about your decision.

**How will participating in the study affect my life?**

You will need to come for regular follow-up visits to have the effectiveness and safety of your treatment evaluated. These follow-up visits are required as part of your clinical practice and this study does not add to the frequency of your additional visits. You may find these visits and examinations inconvenient and require special arrangements. In addition, some of the tests may make you feel uncomfortable. If you have any questions about the tests and procedures in the study you can ask your study doctor.

If you have taken a medication that is prohibited by the study before participating in the study, you will need to stop taking the medication for 24 weeks before you can participate in our study. If you need to stop taking your medication, you will need to talk to your study doctor about how to stop it for your safety.

If you are a woman of childbearing potential, you will be required to use contraception throughout the study, either condoms or ligation. Please consult with your study physician to determine which method of contraception to use and for how long. Certain forms of contraception are not approved during the study and pharmaceutical contraception is not recommended.

You will not be able to participate in any other clinical studies of drugs or medical devices during the entire study period.

**Stored specimens and future research**

(1) Collected specimens will be preserved for future research. Specimens may be stored at all research centers. Transportation of specimens from one research center to another will be done through a secure courier service provider. We hope that these specimens will help us learn more about TB. These research tests we will be performing are different from routine medical tests. Therefore, we will not put the test results into your medical record; however, someone from the research team will discuss the results with you if you ask.

(2) Labeling of stored specimens: We will use a code to label stored specimens that only the research team can use to contact you personally. Your data and specimens will be stored under a research identification number (I D) (not your name). Any data that can be linked to you personally as private information will only be stored to the extent permitted by law.

(3) FUTURE STUDIES: With your consent, we may use your specimens in future studies. The research team will not send your specimen to any investigator not listed in the protocol of a new trial requesting the use of your stored specimen without the approval of the Ethics Committee. An ethics committee is a committee that oversees medical research to protect the rights and welfare of participants. If the specimen is sent outside of the research organization, the specimen will be identified only by a study number. Your name or initials will not be included. The study team may also share information such as your gender, age, health history, or ethnicity with researchers outside the research center.

Researchers will use your specimen for research purposes only. We will not sell your specimen. Future research using your specimen may result in new products, but you will not be paid for these products. Some future studies may require obtaining health information (such as smoking history or current health status) that we do not already have. In such cases, the research team may contact you to obtain this information.

(4) Risks of Stored Specimens: The greatest risk of allowing us to store your specimens would be the inadvertent disclosure of your identifying information (as the information is leaked from the specimen storage database). Because your name is not stored in the database, the chances of this happening are very low.

(5) Benefits of stored specimens: Generally, research using your specimen in the future will not help you, but it can help us learn more about TB.

(6) In addition to routine sputum and blood tests, other specimens may be collected with your consent, including: 1) exhaled breath, which is used to analyze the metabolic composition of exhaled breath related to tuberculosis and may guide your anti-tuberculosis treatment; and 2) urine and feces, which may be used for tuberculosis histology studies. If you do not agree to keep specimens of exhaled breath, urine, and feces, it does not affect your participation in this study. But again, as mentioned earlier, sputum and blood specimens need to be retained. If you have any questions about the tests and steps in the study you can ask the study doctor.

**What are the potential risks to me of participating in this study?**

(1) Regarding the risk of a shorter course of treatment: this study is focused on exploring the shortening and optimization of the course of treatment, and the risk of TB relapse due to a shorter course of treatment does exist, but based on the trends in the field and our prior experience, this risk is low. It is important to note that the risk of relapse of multidrug-resistant TB exists even if you do not participate in this study. Specifically, if our assessment of your condition suggests that you may have been able to stop taking your anti-tuberculosis medication by month 6 or 9 of treatment, and you happen to be randomized to the treatment group in which you stopped taking your medication, you may be potentially at risk for possibly developing TB again as a result of the short course of treatment (6-9 months is a few months shorter than the current WHO recommendation of an 18-20 month regimen). If you do develop TB again, you will need to be treated with anti-TB drugs again. But even with complete use of all the standard WHO-recommended treatments, a small number of people may still develop TB, and it is important to understand this. We think the same is true for the short-course regimen treatment group, and a small number of people in this group may still get TB again. If you need to be put on anti-TB treatment again, there is a chance that the drugs you were initially given will not work. We will monitor you closely during and after treatment for any signs that you may develop TB again. If you do develop TB again, we would like you to stay in the study until you have finished taking the medicines in your new regimen. Please tell your doctor if you feel any discomfort during or after treatment.

(2) About the risk of blood draws: Regular blood tests are required as part of routine TB care; this study does not increase the number of blood draws, but may increase the amount of blood drawn each time. This blood will be used to monitor your drug levels, to guide your treatment, or for basic TB research to benefit other patients in the future. There are some slight risks associated with having your blood drawn. You may feel some discomfort while your blood is being drawn. You may have a small bruise or hard lump on your arm at the site of the blood draw, and the skin around the site of the blood draw may become infected, but this risk is very small. You may also feel dizzy or faint. The staff at the hospital or center will watch you closely to ensure that you receive proper medical attention should any of these problems occur.

(3) Risks of medications: The vast majority of medications used in this study are anti-tuberculosis medications recommended by the World Health Organization. Common risks of these anti-tuberculosis medications include loss of appetite, nausea, vomiting, fever, and skin rash. Your doctor will explain these possible adverse effects and their management in detail before starting treatment. We will closely monitor for adverse events and treat them accordingly, making every effort to ensure safety. If a serious adverse event occurs during the study, your treatment may be terminated after evaluation by the researchers.

(4) Other Risks: For your safety, you must tell the study doctor or nurse about all medications you are taking before you start participating in the study. You must also tell the study doctor or nurse first if you need to receive any new medications while participating in the study. In addition, if you are enrolled in any other clinical trials during your participation in the study, you must tell the study doctor or nurse beforehand.

If you experience any adverse reactions during the study, please call your study doctor for advice promptly. Contact information: (1) Shenzhen Third People's Hospital: Dr. Fu Liang, 15989869571. (2) Other units: study leader and study doctor.

You will need to tell your family or close friends that you are participating in a clinical research study and that they can watch for the events described above. If they have questions about your participation in the study, you can tell them how to contact your study doctor.

Your participation in this study will be terminated for any of the following reasons:

1) You request to be withdrawn from the study, or you are unable to return for regular follow-up appointments;

2) Mycobacterium tuberculosis cultures were negative at the time of enrollment;

3) Expressive drug sensitization results suggest sensitivity to rifampicin;

4) You are infected with HIV;

5) You are pregnant or unable to use effective contraception;

6) The doctor or researcher feels that it is no longer in your best interest to participate in the study and that withdrawing from the study is your best option.

During the course of your participation in this study, we will provide you with new information that may affect your willingness to continue participating in the study.

**Did you benefit from participating in this study?**

Participating in this study may, or may not, make your health better.

If your TB is successfully cured at month 6 or 9, your medication duration will be several months shorter than the WHO recommended protocol. In that case, you will probably experience fewer side effects from the medication and the cost of your doctor's visit will be reduced accordingly. Other than that, you will receive no other benefits.

It is our hope that the information and materials obtained through this study will help determine which treatments may be safer and more effective in treating other patients with conditions similar to yours.

**Costs associated with participation in this study?**

Anti-tuberculosis drugs are not provided free of charge and will be paid for by you. This study will not increase the number of your normal follow-up appointments, and you will pay for your own tests, registration fees at follow-up appointments, and transportation costs while you are participating in this study. You will not receive any payment for participating in this study.

**What happens if I am harmed while participating in the study?**

If you do experience study-related damage to your health as a result of participating in this study, please notify the study physicians immediately and they will be responsible for taking appropriate treatment measures for you. No new or unlisted drugs will be used in this study. In the event of an adverse drug reaction, treatment failure, relapse, or death, it will be handled in accordance with clinical practice, and the research organization will not be responsible for the cost of treatment or provide financial compensation. No compensation will be paid by the study site for injuries resulting from medical malpractice or from failure to follow study protocol procedures.

If you drop out of the study in the middle of the study, you will be treated according to clinical practice.

Even if you have signed this informed consent form, you retain all your legal rights.

If the above terms are not acceptable, you may choose not to participate in this study.

**Is my personal information confidential?**

Your medical records will be kept at the hospital and the investigator, research authorities, and ethics committee will be allowed to access your medical records. The government administration will have access to your information as required. Any public reporting of the results of this study will not disclose your personal identity. We will make every effort to protect the privacy of your personal medical information to the extent permitted by law.

Personal and medical information about you will be kept confidential and stored in a safe and secure place. At any time, you may request access to your personal information (e.g., your name and address) and may correct it if necessary.

When you sign this informed consent form, it represents your consent to the use of your personal and medical information for the purposes described above.

**Do I have to participate in the study?**

**Participation in this study is completely voluntary, and** you may refuse to participate in the study or choose to withdraw from the study at any time during the study without any reason. This decision will not affect your future treatment. If you do not participate in this study, or if you leave the study halfway through, there are other alternative treatment medications available.

Again, even if you are already enrolled in the study, you have the right to leave the study at any time during the trial. If you decide to leave this study, please notify your study doctor in advance. For your safety, you may be asked to undergo tests that may be beneficial to protect your health.

**Who do I call if I have any questions or problems?**

While this study is being conducted, you will be cared for by Director Deng Dongfeng (Study Leader at Shenzhen Third People's Hospital) and the Co-Research Leaders. At any time, if you feel that any of your symptoms are causing you any distress, or if you have any problems during the study, please do not hesitate to contact the study leader or any of the other doctors who are taking part in this study (co-study leaders).

For questions about this study or study-related injuries, (1) in the Shenzhen area, please contact Director Deng Dongfeng at 13530027001 or Dr. Fu Liang at 15989869571. (2) outside of Shenzhen, please contact the study leader at your local unit.

For questions about your rights as a participant in the study, please contact the Ethics Committee of Shenzhen No. 3 People's Hospital at 0755-61222333-6665.The Ethics Committee of Shenzhen No. 3 People's Hospital is a committee of professionals responsible for reviewing the study in order to protect the rights of the study participants.

**Research-specific consent statements**

**By signing below, I agree:**

In response to the Multicenter Randomized Withdrawal Trial of an All-Oral Short-Course Regimen for the Treatment of Multidrug-Resistant Tuberculosis, I have read this Informed Consent Form, and I have been given the opportunity to ask questions and have had all of my questions answered.

I have been informed about the purpose, significance and specific methods of this study, and I am aware of the therapeutic efficacy of the treatment regimen, possible adverse effects, etc.

I may choose not to participate in this study or withdraw at any time by notifying the investigator without discrimination or retaliation, and none of my medical treatment or rights will be affected as a result. If I am physically harmed or die as a direct result of a serious adverse reaction directly attributable to the medication used in this study, or if I incur study-related harm or death, the reasonable, usual, and necessary costs of such harm or death will be resolved by negotiation. The investigating physician may terminate my continued participation in this study if I need other treatment or if I do not comply with the study plan or for any other reason.

I agree that my specimen may be stored in a laboratory at a different research center and may only be used for future research.

I will receive a signed copy of the Informed Consent Form. I agree to participate in this study and am willing to cooperate with the investigator as required to complete the clinical study.

**Patient Signature: Date:**

**Patient's name in block letters: Contact phone number:**

**Signature of guardian (if any): Date:**

**Guardian's name in block letters: Contact phone number:**

(Note: Signature of a witness if the subject is illiterate and a guardian if the subject is incapacitated.)

**Researcher Statement:**

I have fully and carefully explained this program to the above participant to the best of my knowledge. I confirm that, to the best of my knowledge, the above participant clearly understands the nature of the program, the risks and benefits of participation.

I confirm that I have given the above participants ample opportunity to ask relevant questions and that I have accurately answered, to the best of my ability, all the questions they have asked.

I confirm that the above participating patient was not coerced into signing the consent form and that he/she did so freely and voluntarily.

I confirm that the above subject has been provided with a signed consent form.

**Signature of researcher: Date:**

**Researcher's name in block letters: Contact phone number:**

**Ethics Committee of Shenzhen Third People's Hospital Tel: 0755-61222333-6665**

**Sub-center name: Ethics Committee Phone**:
